# Morphological and molecular identification of three new bamboo-inhabiting species of *Nigrochaete* (*Auriculariales*, *Basidiomycota*) from China

**DOI:** 10.3897/mycokeys.132.184839

**Published:** 2026-05-15

**Authors:** Qi Yuan, Qiong Chen, Shichun You, Xiangfu Liu, Changlin Zhao

**Affiliations:** 1 Key Laboratory of Forest Disaster Warning and Control in Universities of Yunnan Province, Southwest Forestry University, Kunming 650224, China Department Microbial Drugs (MWIS), Helmholtz-Centre for Infection Research Braunschweig Germany; 2 College of Forestry, Southwest Forestry University, Kunming 650224, China College of Forestry, Southwest Forestry University Kunming China; 3 Department Microbial Drugs (MWIS), Helmholtz-Centre for Infection Research, 38124 Braunschweig, Germany Key Laboratory of Forest Disaster Warning and Control in Universities of Yunnan Province, Southwest Forestry University Kunming China

**Keywords:** Biodiversity, molecular systematics, pairwise homoplasy index, taxonomy, Yunnan Province

## Abstract

*Nigrochaete* is a small fungal genus responsible for bamboo rot. Although recent research has clarified its classification through integrated phylogenetic and morphological analyses, the species diversity of *Nigrochaete* in the high-altitude forests of Yunnan Province, China, remains insufficiently explored. This study collected five *Nigrochaete* specimens from decaying bamboo in the Dehong Dai and Jingpo Autonomous Prefecture and examined their morphological characteristics. Genomic DNA was extracted, and the internal transcribed spacer (ITS), nuclear large subunit ribosomal DNA (nrLSU), and translation elongation factor 1-alpha (*TEF*1) gene were amplified and sequenced. Phylogenetic relationships within *Nigrochaete* were inferred using maximum likelihood (ML) and Bayesian inference (BI) methods. These analyses identified three novel species: *N. albobadia*, *N. ellipsoidea*, and *N. tenuis*. Detailed morphological descriptions, micrographs, and phylogenetic results are presented. This research expands the known species diversity of *Auriculariales* in China and provides a foundation for the conservation and sustainable utilization of fungal resources.

## Introduction

Wood-inhabiting fungi represent a highly diverse group of microorganisms that play essential roles in forest ecosystems; they are considered key players in wood decomposition because of their ability to produce lignocellulosic enzymes that break down woody lignin, cellulose, and hemicellulose (Tedersoo et al. 2014; Schoutteten et al. 2024; Case et al. 2025; Yuan et al. 2026).

The order *Auriculariales* is mainly composed of wood-inhabiting fungi within *Agaricomycetes* Doweld (*Basidiomycota*) (Hibbett et al. 2007). The type genus *Auricularia* Bull. includes several economically important edible and medicinal species, as well as other gelatinous genera such as *Exidia* Fr., *Guepinia* Fr., and *Pseudohydnum* P. Karst. (Liu et al. 2022). Therefore, interest in species diversity in gelatinous genera has increased significantly in recent years (Hibbett et al. 2014; Wu et al. 2021; Tohtirjap et al. 2023; Dong et al. 2024). In contrast to the gelatinous genera, most species in the order *Auriculariales* are tough, including saprophytic species with resupinate, effused-reflexed, hydnoid, cerebriform, coralloid, or pileate basidiomata (Wells and Bandoni 2001; Miettinen et al. 2012; Hibbett et al. 2014; Malysheva and Spirin 2017; Alvarenga et al. 2019; Spirin et al. 2019a, 2019b; Tohtirjap et al. 2023). Species with stereoid basidiocarps are widely distributed in many orders of *Agaricomycetes*, although they are certainly a minority in the order *Auriculariales* (Malysheva and Spirin 2017; Dong et al. 2024).

The genus *Nigrochaete* J.H. Dong & C.L. Zhao was erected and typified by *N. bambusicola* J.H. Dong & C.L. Zhao (Dong et al. 2025a). It is characterized by membranaceous basidiomata, grandinoid, occasionally cracked hymenophore, a monomitic hyphal system with clamp connections on generative hyphae, cystidia and hyphidia present, subellipsoid to ovoid basidia, and allantoid, slightly curved basidiospores (Dong et al. 2025a). According to the MycoBank database (http://www.mycobank.org, accessed on 17 April 2026) and Index Fungorum (http://www.indexfungorum.org, accessed on 17 April 2026), *Nigrochaete* is currently a monotypic genus, comprising only the type species *N. bambusicola* (Dong et al. 2025a).

Currently, molecular phylogenetic analyses based on multiple genes play a vital role in the taxonomy of *Auriculariales*, particularly within *Auriculariaceae*, not only in the delineation of novel species but also in revising the classification of currently unclassified taxa (Weiss and Oberwinkler 2001; Malysheva et al. 2018). Weiss and Oberwinkler (2001) constructed phylogenetic relationships in *Auriculariales* and related groups based on nuclear large subunit ribosomal DNA (nrLSU) sequences, proposing that *Auriculariales* was a polyphyletic group and that the family *Sebacinaceae* was confirmed as a monophyletic group, which appeared distant from other taxa ascribed to *Auriculariales* (Weiss and Oberwinkler 2001). Wells et al. (2004) reconsidered the families *Auriculariaceae* and *Hyaloriaceae* and considered *Exidiaceae* a synonym of *Auriculariaceae*. *Exidia* species were also classified into *Auriculariaceae* by several researchers (Zhou and Dai 2013; Yuan et al. 2018; Spirin et al. 2019a, 2019b; He et al. 2024). *Hyaloriaceae* was rarely mentioned and even abandoned (He et al. 2024). However, based on internal transcribed spacer (ITS)+nrLSU phylogenetic analyses, the phylogenetic clade included *Myxarium* species and *Hyaloria pilacre*, which was distant from other core taxa of *Auriculariaceae* (Weiss and Oberwinkler 2001). Tohtirjap et al. (2023) reached the same conclusion through combined ITS+nrLSU phylogenetic analyses. Therefore, this family was accepted by some researchers (Wells et al. 2004; Tohtirjap et al. 2023). Currently, two families, *Auriculariaceae* and *Hyaloriaceae*, are accepted in the order (He et al. 2024; Spirin et al. 2025). In recent years, the species diversity of the resupinate *Auriculariales* has been described or better defined using morphological and molecular analyses, and the results showed the hidden diversity of this group and several corticioid genera, for example, *Adustochaete*, *Alloexidiopsis*, *Amphistereum* Spirin & Malysheva, *Crystallodon* Alvarenga, *Heterocorticium*, *Heteroradulum*, *Metulochaete* Alvarenga, *Nodulochaete* J.H. Dong & C.L. Zhao, *Proterochaete* Spirin & Malysheva, *Punctochaete* J.H. Dong & C.L. Zhao, and *Sclerotrema* Spirin & Malysheva, which have been established and described based on morphological and phylogenetic studies (Malysheva and Spirin 2017; Alvarenga et al. 2019; Spirin et al. 2019a, 2019b; Alvarenga and Gibertoni 2021; Dong et al. 2025c). Based on phylogenetic analyses of combined ITS and nrLSU sequences, the study proposed the new genus *Nigrochaete*, with *Nigrochaete bambusicola* designated as the type species (Dong et al. 2025a). However, many species within this genus remain undiscovered.

During investigations of wood-inhabiting fungi in Yunnan Province, China, five specimens were collected. To clarify the placement and relationships of these specimens, a phylogenetic and taxonomic study was conducted based on ITS+nrLSU+*TEF*1 sequences. These specimens were assigned to the genus *Nigrochaete* within the order *Auriculariales*, and three new species, *N. albobadia*, *N. ellipsoidea*, and *N. tenuis*, are proposed, with descriptions and illustrations based on morphological characteristics and phylogenetic analyses. Additionally, pairwise homoplasy index (PHI) analyses were conducted for the three new species and their similar taxa.

## Materials and methods

### Sample collection and herbarium specimen preparation

The fresh fruiting bodies were collected on dead bamboo from Dehong Dai and Jingpo Autonomous Prefecture, Yunnan Province, China. The samples were photographed *in situ* with a Nikon D7100 camera, and information on collection was noted (Rathnayaka et al. 2025), along with fresh macroscopic details. All photographs were focus-stacked using Helicon Focus software. Collections were transported to the field station in plastic mushroom collection boxes, where the fruiting bodies were dried on an electronic food dryer at 45 °C (Dong et al. 2025b). Once dried, the specimens were sealed in envelopes and zip-lock plastic bags and labeled (Yang et al. 2025). The dried specimens were deposited in the Herbarium of Southwest Forestry University (SWFC), Kunming, Yunnan Province, China.

### Morphology

The macro-morphological descriptions were based on field notes and photographs collected in the field and in the laboratory. Petersen (1996) was followed for color terminology. The micro-morphological data were obtained from dried specimens observed under a light microscope at 1000× oil immersion (Zhao et al. 2023; Dong et al. 2025b). Sections were mounted in 5% KOH and 1% phloxine B (C_20_H_2_Br_4_Cl_4_Na_2_O_5_), and Cotton Blue and Melzer’s reagent were used where necessary to observe micromorphology following the method of Yang et al. (2025). To present the variations in spore sizes, 5% of measurements were excluded from each end of the range and shown in parentheses. A minimum of 30 basidiospores from each specimen was measured. Stalks were excluded from basidia measurements, and the hilar appendage was excluded from basidiospore measurements. The MycoBank numbers were registered in the MycoBank database (http://www.mycobank.org).

The following abbreviations are used: **CB** = Cotton Blue, **CB+** = cyanophilous, **CB–** = acyanophilous, **IKI** = Melzer’s reagent, **IKI–** = both inamyloid and nondextrinoid, **KOH** = 5% potassium hydroxide water solution, ***L*** = mean spore length (arithmetic average for all spores), ***W*** = mean spore width (arithmetic average for all spores), ***n*** = a/b (number of spores (a) measured from the given number (b) of specimens), and ***Q*** = variation in the *L*/*W* ratios between the specimens studied.

### Molecular phylogeny

The CTAB rapid fungi genome extraction kit-DN14 (Aidlab Biotechnologies Co., Ltd., Beijing) was used to obtain genomic DNA from dried specimens according to the manufacturer’s instructions. The gene fragments employed in this study are detailed in Table 1.

**Table 1.** Gene regions, respective primers and references used in the study.

Gene region	Primer pairs	Sequence (5’-3’)	Annealing temperature (°C)	References
ITS	ITS5	GGA AGT AAA AGT CGT AAC AAG G	55 °C	White et al. (1990)
	ITS4	TCC TCC GCT TAT TGA TAT GC		
nrLSU	LR0R	ACC CGC TGA ACT TAA GC	48 °C	Vilgalys and Hester (1990)
	LR7	TAC TAC CAC CAA GAT CT		
*TEF*1	EF1-983F	GCY CCY GGH CAY CGT GAY TTY AT	59 °C	Rehner and Buckley (2005)
	EF1-2218R	ATG ACA CCR ACR GCR ACR GTY TG		

The PCR protocol for ITS was as follows: initial denaturation at 95 °C for 3 min, followed by 35 cycles at 94 °C for 40 s, and 55 °C for 40 s. The PCR protocol for nrLSU and TEF1 was as follows: initial denaturation at 94 °C for 1 min, followed by 35 cycles at 94 °C for 30 s, 48 °C for nrLSU and 59 °C for TEF1 for 1 min, and 72 °C for 1.5 min, and a final extension of 72 °C for 10 min (Dong et al. 2025b; Deng et al. 2026). The PCR products were purified and sequenced at Kunming Tsingke Biological Technology Limited Company, Kunming, Yunnan Province, P.R. China. All newly generated sequences were deposited in GenBank (Table 2).

**Table 2.** List of species, specimens, and GenBank accession numbers of sequences used in this study.

Species name	Sample no.	GenBank accession no.			Country	References
		ITS	nrLSU	*TEF*1		
*Adustochaete nivea*	RLMA 531	MN165954	MN165989	—	Brazil	Alvarenga et al. (2019)
*Adustochaete rava*	KHL 15526	MK391517	MK391526	—	Brazil	Alvarenga et al. (2019)
*Alloexidiopsis australiensis*	LWZ 20180514-18	OM801934	OM801919	—	China	Liu et al. (2022)
*Alloexidiopsis schistacea*	LWZ 20200819-21a	OM801939	OM801932	—	China	Liu et al. (2022)
*Amphistereum leveilleanum*	FP-106715	KX262119	KX262168	—	USA	Malysheva and Spirin (2017)
*Amphistereum schrenkii*	HHB 8476	KX262130	KX262178	—	USA	Malysheva and Spirin (2017)
*Aporpium caryae*	WD 2207	AB871751	AB871730	—	Japan	Sotome et al. (2014)
*Auricularia heimuer*	xiaoheimao	KM396781	KM396834	—	China	Wang et al. (2023)
*Auricularia mesenterica*	Kytovuori-89-333	KP729284	KP729302	—	Estonia	Wu et al. (2021)
*Basidiodendron luteogriseum*	KHL 16022	MT040881	MT040861	—	Brazil	Spirin et al. (2020)
*Basidiodendron trachysporum*	VS 11886	MW152419	MW136128	—	Russia	Spirin et al. (2021)
*Bourdotia galzinii*	Otto MiettinenX3067	MG757511	MG757511	—	Spain	Malysheva et al. (2018)
*Crystallodon subgelatinosum*	RC1609	MN475884	MN475888	—	Brazil	Alvarenga and Gibertoni (2021)
*Ductifera pululahuana*	KW 1733	—	AF291315	—	USA	Weiss and Oberwinkler (2001)
*Eichleriella alliciens*	HHB 7194	KX262120	KX262169	—	USA	Malysheva and Spirin (2017)
*Eichleriella alpina*	He 20120916-1	MH178245	MH178268	—	China	Li et al. (2023)
*Elmerina cladophora*	X1902	MG757509	MG757509	—	Indonesia	Malysheva et al. (2018)
*Endoperplexa dartmorica*	VS 11781	MT235621	MT235602	—	Norway	Spirin et al. (2025)
*Exidia glandulosa*	Dai 21232	MT663362	MT664781	—	China	Wu et al. (2020)
*Exidia pithya*	MW 313	AF291275	AF291321	—	Germany	Weiss and Oberwinkler (2001)
*Exidiopsis effusa*	Miettinen 19136	KX262145	KX262193	—	Finland	Tohtirjap et al. (2023)
*Gelacantha pura*	LE 254018	MK098882	MK098930	—	Russia	Spirin et al. (2019a)
*Grammatus labyrinthinus*	Yuan 1600	KM379139	KM379140	—	China	Alvarenga et al. (2019)
*Grammatus semis*	OM10618	KX262146	KX262194	—	China	Malysheva and Spirin (2017)
*Guepinia montana*	Yang6743	OR822233	OR803013	—	China	Cui et al. (2024)
*Helicomyxa everhartioides*	TNM	—	AY640107	—	China	Kirschner and Chen (2004)
*Heterochaete andina*	Lagerheim	—	KX262187	—	Ecuador	Malysheva and Spirin (2017)
*Heterocorticium bambusicola*	He4604	MH178260	MH178284	—	China	Li et al. (2023)
*Heterocorticium latisporum*	He20120923-20	MH178261	MH178285	—	China	Li et al. (2023)
*Heteroradulum adnatum*	LR 23453	KX262116	KX262165	—	Mexico	Malysheva and Spirin (2017)
*Heteroradulum kmetii*	VS 6466	KX262104	KX262152	—	Russia	Malysheva and Spirin (2017)
*Hyalodon antui*	Niemelä 6389	MG735416	MG735424	—	China	Spirin et al. (2019b)
*Hyalodon piceicola*	Spirin 11063	MG735415	MG735423	—	Russia	Spirin et al. (2019b)
*Hyaloria pilacre*	TI 2768	—	AF291338	—	Venezuela	Weiss and Oberwinkler (2001)
*Hydrophana sphaerospora*	VS 11622	MK098884	MK098932	—	Norway	Spirin et al. (2019a)
*Hydrophana sphaerospora*	VS 11133	MK098883	MK098931	—	Norway	Spirin et al. (2019a)
*Metulochaete sanctae-catharinae*	AM 0678	MK484065	MK480575	—	Russia	Spirin et al. (2019b)
*Mycostilla vermiformis*	Spirin 11330	MG735417	MG735425	—	Russia	Spirin et al. (2019b)
*Myxariellum concinnum*	VS 8393c	MK098885	MK098933	—	USA	Spirin et al. (2019a)
*Myxarium nucleatum*	Spirin 10013	KY801879	KY801906	—	Norway	Spirin et al. (2018)
***Nigrochaete albobadia***	**CLZhao 43229**	**PX831828**	**PX837119**	**PZ349602**	**China**	**Present study**
***Nigrochaete albobadia***	**CLZhao 44349**	**PX831829**	**PX837120**	**PZ363740**	**China**	**Present study**
*Nigrochaete bambusicola*	CLZhao 11314	PQ571173	PQ571168	—	China	Dong et al. (2025b)
*Nigrochaete bambusicola*	CLZhao 11307	PQ571172	PQ571167	—	China	Dong et al. (2025b)
***Nigrochaete ellipsoidea***	**CLZhao 45047**	**PX831832**	—	**PZ363742**	**China**	**Present study**
***Nigrochaete tenuis***	**CLZhao 44159**	**PX831830**	**PX837121**	**PZ363739**	**China**	**Present study**
***Nigrochaete tenuis***	**CLZhao 44987**	**PX831831**	**PX837122**	**PZ363741**	**China**	**Present study**
*Nodulochaete fssurata*	CLZhao 27533	PQ166573	PQ166565	—	China	Dong et al. (2025c)
*Nodulochaete punctata*	CLZhao 22803	PQ166569	PQ166561	—	China	Dong et al. (2025c)
*Ofella glaira*	VS 11809	MK098920	MK098964	—	Norway	Spirin et al. (2019a)
*Ovipoculum album*	HKAS57058	—	GU292813	—	China	Kirschner et al. (2010)
*Porpopycnis lubae*	R. Kirschner et al. 2745 (M)	—	HQ914244	—	Panama	Kirschner et al. (2012)
*Proterochaete adusta*	VS 9021	MK391520	MK391528	—	Canada	Alvarenga et al. (2019)
*Protoacia delicata*	VS 4615	MK098923	MK098967	—	Russia	Spirin et al. (2019a)
*Protodaedalea hispida*	Spirin 5139	MG757510	MG757510	—	Finland	Spirin et al. (2019b)
*Protodontia africana*	AS 171126/1104	MK098978	MK098973	—	Russia	Spirin et al. (2019a)
*Protohydnum cartilagineum*	SP 467240	MG735419	MG735426	—	Russia	Spirin et al. (2019b)
*Protomerulius brasiliensis*	Ryv. 19735	—	AF291359	—	Argentina	Weiss and Oberwinkler (2001)
*Protomerulius substuppeus*	O 19171	JX134482	JQ764649	—	China	Zhou and Dai (2013)
*Pseudohydnum gelatinosum*	AFTOL ID1875	DQ520094	DQ520094	—	Germany	Lutzoni et al. (2004)
*Psilochaete multifora*	VS 11596	MK484066	MK480576	—	Norway	Spirin et al. (2019b)
*Punctochaete murina*	CLZhao 32118	PP819689	PP819701	—	China	Dong et al. (2025d)
*Renatobasidium notabile*	VS 11118	MT235654	MT353648	—	Norway	—
*Sclerotrema griseobrunnea*	VS 7674	KX262140	KX262188	—	Russia	Malysheva and Spirin (2017)
*Sistotrema brinkmannii*	isolate 236	JX535169	JX535170	—	Netherlands	Alvarenga and Gibertoni (2021)
*Stypella papillata*	KHL 11751	EU118672	—	—	Finland	Larsson (2007)
*Stypellopsis farlowii*	Larsson 12337	MG857095	MG857099	—	Russia	Spirin et al. (2019c)
*Stypellopsis hyperborea*	J Norden 9751	MG857097	MG857101	—	Russia	Spirin et al. (2019c)
*Tremellochaete atlantica*	URM90199	MG594381	MG594383	—	Brazil	Alvarenga et al. (2019)

Note: “–” indicates data unavailability; newly generated sequences are represented in bold.

Phylogenetic analyses followed the methods in Dissanayake et al. (2020). Newly generated sequence data were initially subjected to a BLAST search in NCBI to obtain the most probable closely related taxa in GenBank (http://blast.ncbi.nlm.nih.gov/). Sequence data were retrieved from GenBank based on recent publications (https://www.ncbi.nlm.nih.gov/nuccore/). The sequences were aligned using MAFFT version 7 (Katoh et al. 2019) with the G-INS-I strategy. The alignment was adjusted manually using AliView version 1.27 (Larsson 2014). The dataset was initially aligned, and later, ITS, nrLSU and *TEF*1 sequences were combined using Mesquite version 3.51. FASTA data file formats were converted to PHYLIP and NEXUS formats using the online tool available on the ALTER website (http://sing.ei.uvigo.es/ALTER/, Glez-Peña et al. 2010). Phylogenetic trees were constructed based on randomized accelerated maximum likelihood (ML) and Bayesian inference (BI) analyses.

Pairwise genetic distances (*p*-distance) between *Nigrochaete* species were calculated using the ITS dataset in MEGA 7. All positions containing gaps and missing data were eliminated (Jeewon and Hyde 2016). Maximum likelihood analyses were performed using the CIPRES Science Gateway (https://www.phylo.org/portal2/login!input.action; Miller et al. 2012) based on the dataset using the RAxML-HPC BlackBox tool, with RAxML halting bootstrapping automatically and 0.25 for maximum hours and obtaining the best tree using ML search. Other parameters in ML analysis used default settings, and statistical support values were obtained using nonparametric bootstrapping with 1000 replicates. Bayesian inference analysis was performed on the same dataset using MrBayes v3.2.7a (Ronquist et al. 2012). The best substitution model for the dataset was selected using ModelFinder v2.2.0 (Kalyaanamoorthy et al. 2017) with the Bayesian information criterion, and the model was used for Bayesian analysis. Four Markov chains were run from random starting trees. Trees were sampled every 1000^th^ generation. The first 25% of sampled trees were discarded as burn-in, while the remaining trees were used to construct a 50% majority consensus tree and to calculate Bayesian posterior probabilities (BPPs).

Phylogenetic trees were visualized and adjusted using FigTree v1.4.4 (http://tree.bio.ed.ac.uk/software/figtree), and the exports were edited using Adobe Illustrator CS6 software (Adobe Systems, USA). Branches of the consensus tree received bootstrap support for ML equal to or above 70% and BI equal to or above 0.9, respectively.

## Results

### Pairwise genetic distances

The pairwise sequence divergence in the ITS region among the three new species and their relatives is shown in Table 3. The genetic distances between *Nigrochaete bambusicola* and the new species *N. albobadia*, *N. ellipsoidea*, and *N. tenuis* were 0.027, 0.027, and 0.033, respectively. Furthermore, the interspecific genetic distances among the three new species (*N. albobadia*, *N. ellipsoidea*, and *N. tenuis*) were all greater than 0.015. For practical purposes, a minimum of > 1.5% nucleotide differences in the ITS region may be indicative of a new species (Jeewon and Hyde 2016). The values observed in the target group strictly meet or significantly exceed this recognized interspecific threshold, firmly supporting the recognition of these taxa as distinct species.

**Table 3.** Pairwise genetic distances for the ITS gene of members of *Nigrochaete*.

Species name	1	2	3
*Nigrochaete bambusicola* CLZhao 11314			
***Nigrochaete albobadia* CLZhao 43229**	0.027		
***Nigrochaete ellipsoidea* CLZhao 45047**	0.027	0.035	
***Nigrochaete tenuis* CLZhao 44159**	0.033	0.023	0.038

Note: New species are represented in bold.

### Phylogenetic analyses

**Figure 1.**
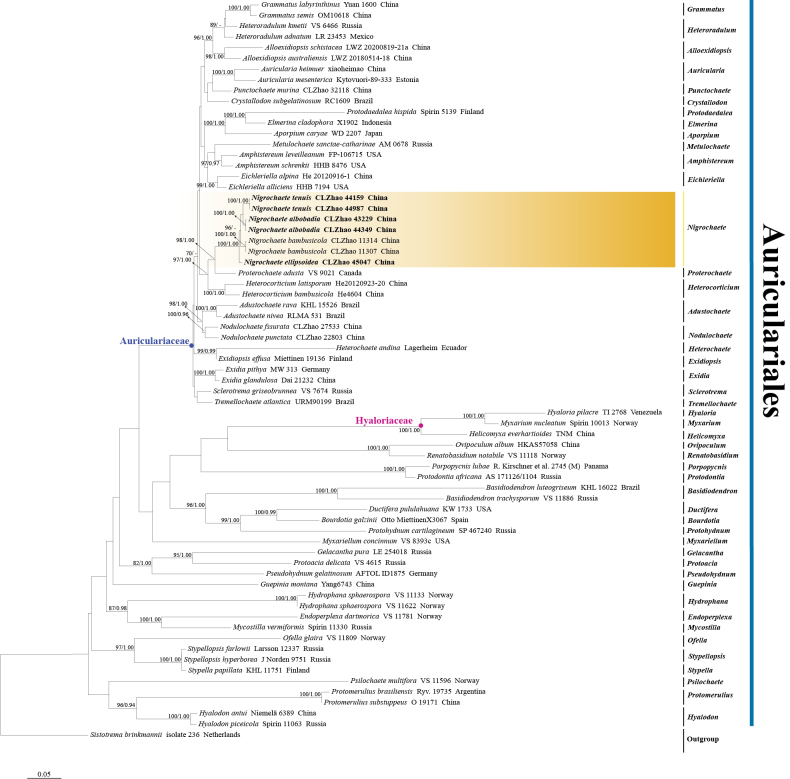
Maximum likelihood phylogenetic tree of *Nigrochaete* and related genera in the order *Auriculariales*, based on the ITS+nrLSU sequence dataset. Branches are labeled with ML bootstrap values equal to or above 70% and BI posterior probabilities equal to or above 0.9. The new species are in bold.

The combined ITS+nrLSU dataset (Fig. 1) included sequences from 69 fungal specimens representing 65 species, and *Sistotrema brinkmannii* (Bres.) J. Erikss. was retrieved as the outgroup taxon (Dong et al. 2025b). A total of four Markov chains were run for two independent runs from random starting trees, each with 1.85 million generations for the ITS+nrLSU dataset, with trees and parameters sampled every 1000 generations. ModelFinder v2.2.0 (Kalyaanamoorthy et al. 2017) was used to select the best-fit model using the BIC criterion. The best model for the combined ITS+nrLSU dataset, as estimated and applied in the Bayesian analysis, was GTR+I+G. Maximum likelihood (ML) and Bayesian inference (BI) analyses yielded similar topologies, with an average standard deviation of split frequencies = 0.009852 (BI) and an effective sample size (ESS; avg. ESS) = 699.5.

**Figure 2.**
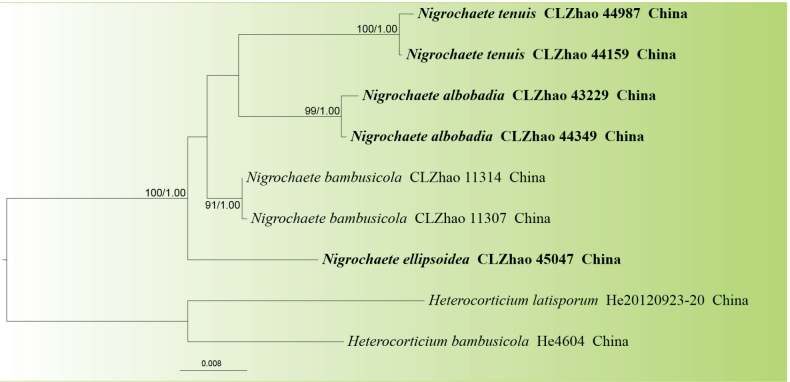
Maximum likelihood phylogenetic tree of the three new species and related species in the genus *Nigrochaete*, based on the ITS+nrLSU+*TEF*1 sequence dataset. Branches are labeled with ML bootstrap values equal to or above 70% and BI posterior probabilities equal to or above 0.9. The new species are in bold.

The ITS+nrLSU+*TEF*1 dataset (Fig. 2) included sequences from nine fungal specimens representing six species. Sequences of *Heterocorticium bambusicola* S.H. He, T. Nie & Yue Li and *H. latisporum* S.H. He, T. Nie & Yue Li were retrieved from GenBank and used as the outgroup (Dong et al. 2025a). A total of four Markov chains were run for two independent runs from random starting trees, each with 1 million generations for the ITS+nrLSU+*TEF*1 dataset, with trees and parameters sampled every 1000 generations. ModelFinder v2.2.0 (Kalyaanamoorthy et al. 2017) was used to select the best-fit model using the BIC criterion. The best model for the combined ITS+nrLSU+*TEF*1dataset, as and applied in the Bayesian analysis was GTR+I+G. ML and BI analyses yielded similar topologies, with an average standard deviation of split frequencies = 0.002511 (BI) and an effective sample size (ESS; avg. ESS) = 754.

Phylogenetic analyses based on the combined ITS+nrLSU dataset (Fig. 1) revealed that three new species, *Nigrochaete albobadia*, *N. ellipsoidea*, and *N. tenuis*, are nested within the genus *Nigrochaete* in *Auriculariales*. In the ITS+nrLSU+*TEF*1-based phylogeny (Fig. 2), *N. albobadia* is sister to *N. tenuis* and forms a well-supported clade, whereas *N. ellipsoidea* constitutes an independent lineage closely related to *N. bambusicola*.

**Figure 3.**
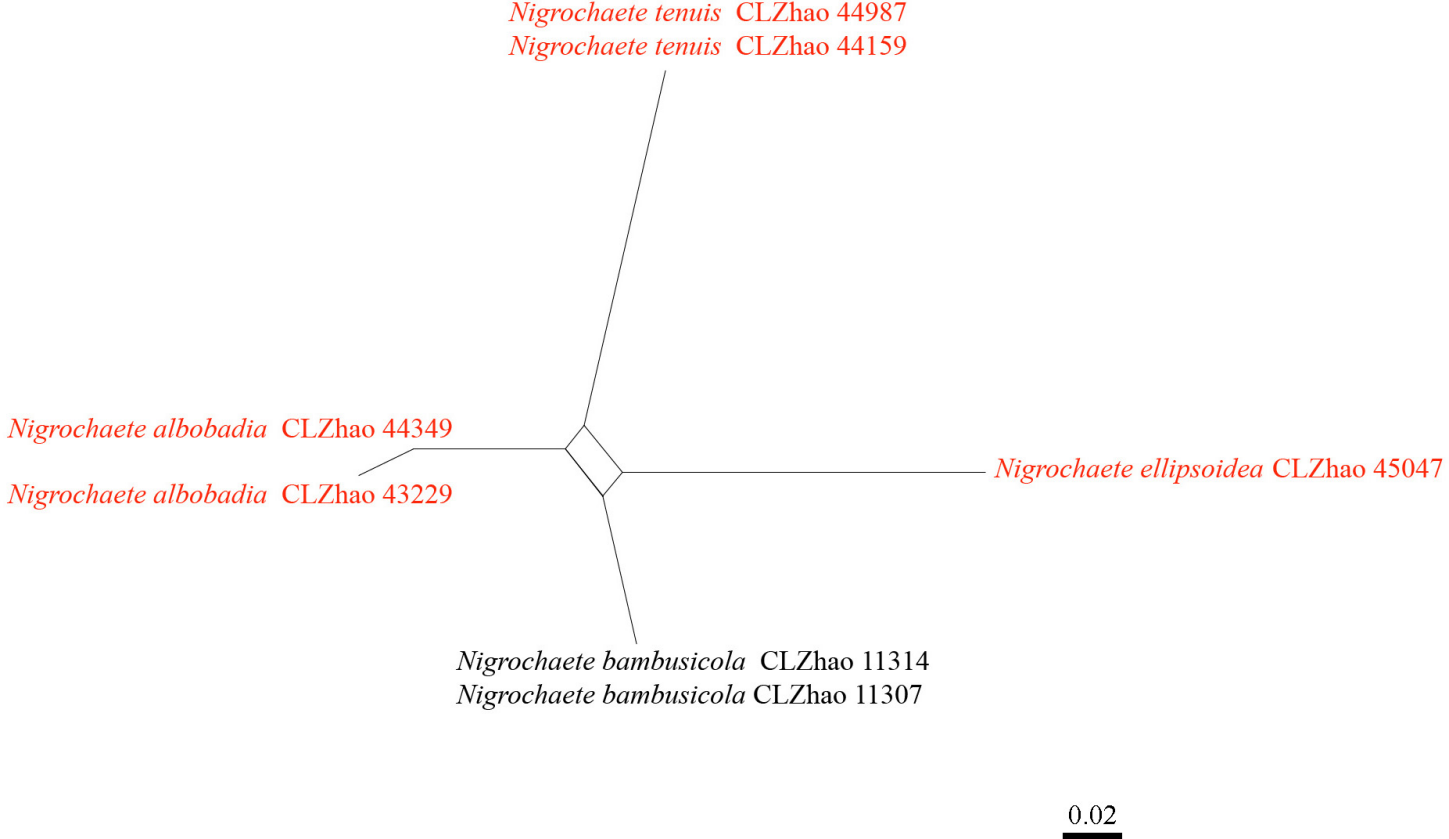
Split graphs showing the results of the PHI test for the ITS data of *Nigrochaete albobadia*, *N. ellipsoidea*, and *N. tenuis* and closely related taxa using LogDet transformation and splits decomposition. PHI test results (Φw ≤ 0.05) indicate significant recombination within the dataset. New taxa are in red.

The application of the PHI test to the ITS tree-locus sequences revealed no evidence of recombination among phylogenetically related species. No significant recombination events were observed among *Nigrochaete albobadia*, *N. ellipsoidea*, and *N. tenuis* and the phylogenetically closely related species *N. bambusicola* (Fig. 3). The PHI test conducted on the ITS sequence alignment (525 bp, containing 13 parsimony-informative sites) yielded a value of Φw = 0.2589 (Φw > 0.05), indicating that no recombination is present in the three new species with *N. bambusicola*.

### Taxonomy

#### Nigrochaete albobadia

Taxon classificationFungiAuricularialesBasidiomycota

Q. Yuan & C.L. Zhao sp. nov.

717D7CFA-809B-56BC-A30D-D7037F1B8DDE

861884

Figs 4, 5, 6

##### Diagnosis.

It is characterized by its membranaceous, thin basidiomata with white, smooth hymenial surface, a monomitic hyphal system with clamped generative hyphae, and allantoid to subcylindrical basidiospores measuring 7.5–9.5 × 4–5 µm.

**Figure 4.**
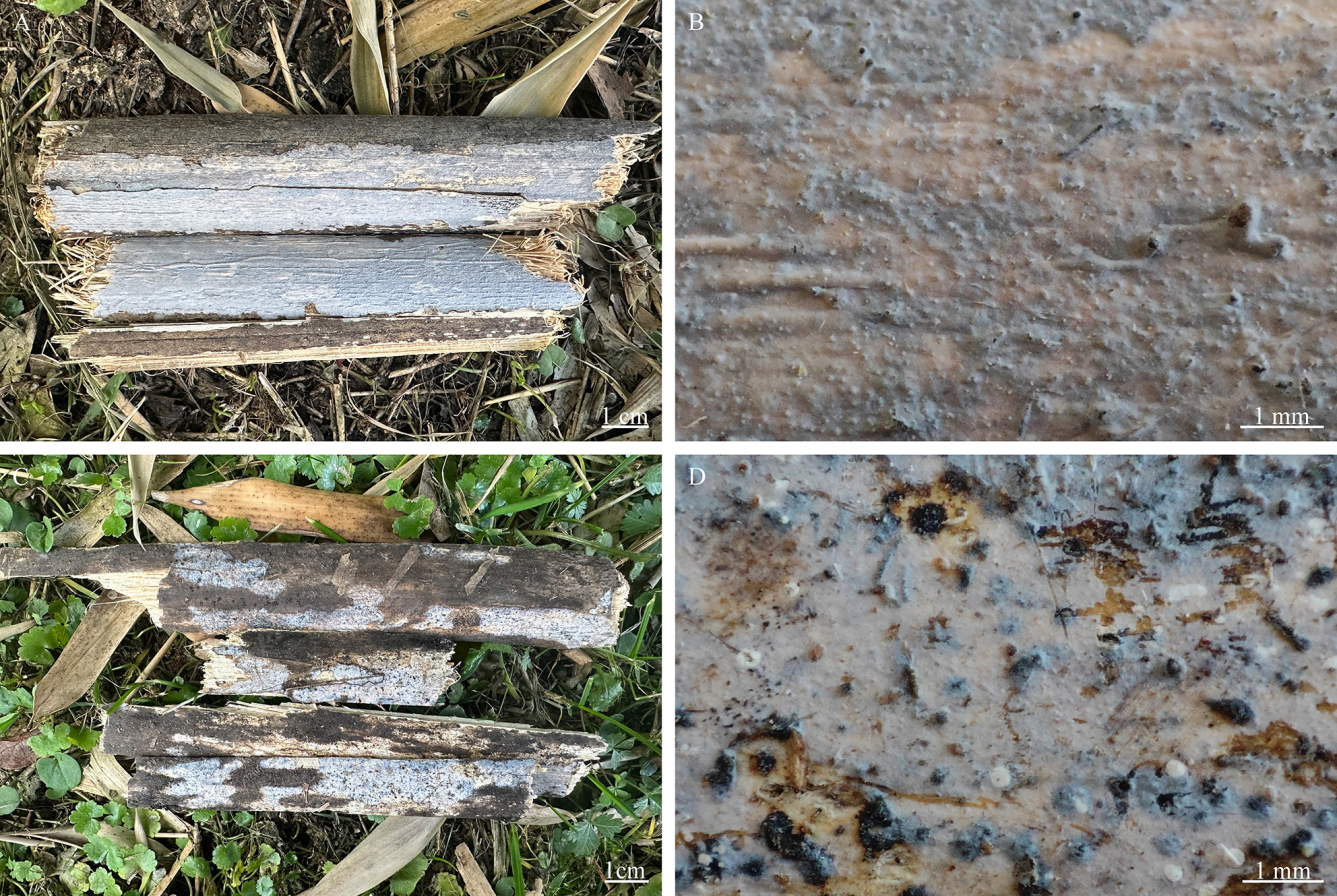
Basidiomata of *Nigrochaete albobadia* (CLZhao 43229, holotype). **A, B**. CLZhao 43229; **C, D**. CLZhao 44349.

**Figure 5.**
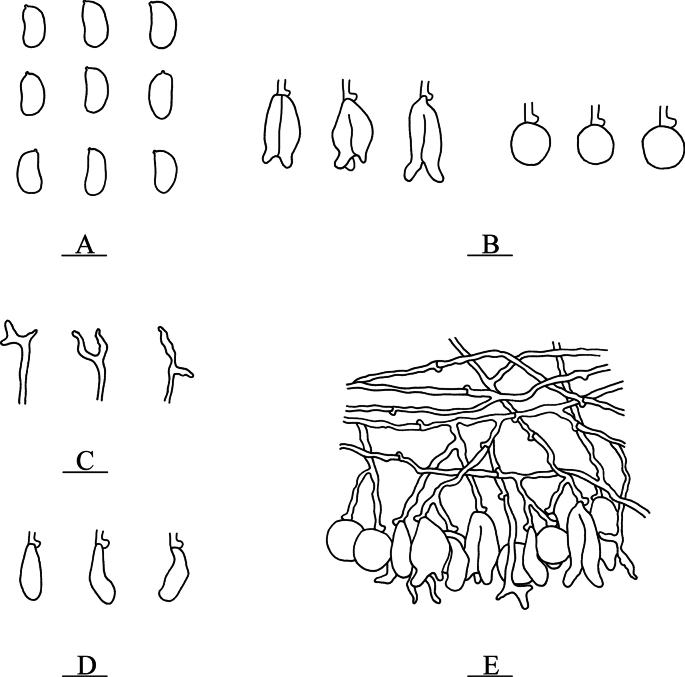
Microscopic structures of *Nigrochaete albobadia* (CLZhao 43229, holotype). **A**. Basidiospores; **B**. Basidia and basidioles; **C**. Hyphidia; **D**. Cystidia; **E**. Part of the vertical section of the hymenium. Scale bars: 10 µm (**A–E**).

**Figure 6.**
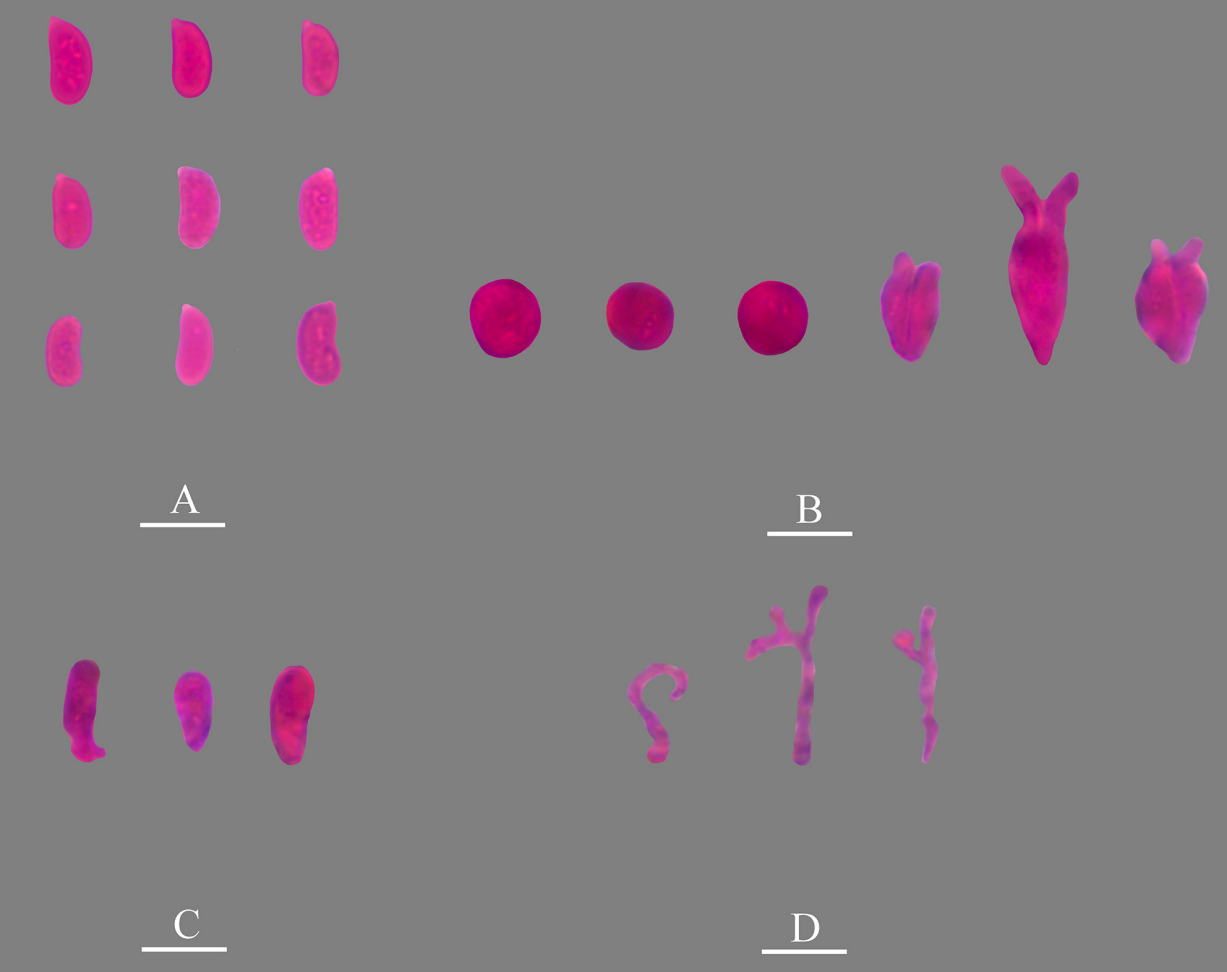
Sections of the hymenium of *Nigrochaete albobadia* (CLZhao 43229, holotype). **A**. Basidiospores; **B**. Basidia and basidioles; **C**. Cystidia; **D**. Hyphidia. Scale bars: 10 µm (**A–D**); 10 × 100 oil.

##### Holotype.

China • Yunnan Province, Dehong, Ruili City, Tongbiguan Provincial Nature Reserve, GPS coordinates 24°25'N, 97°45'E, altitude 2350 m asl., on the dead bamboo, leg. C.L. Zhao, 25 November 2024, CLZhao 43229 (SWFC 00043229).

##### Etymology.

albobadia (Lat.) refers to the white basidiomata of the type species.

##### Basidiomata.

Annual, resupinate, membranaceous, thin, closely adnate, without odor or taste when fresh, up to 12.5 cm long, 2 cm wide, and up to 50 µm thick. Hymenial surface smooth, white when fresh and drying. Sterile margin narrow, white, up to 1 mm.

##### Hyphal system.

Monomitic, generative hyphae with clamp connections, colorless, thin-walled, smooth, interwoven, 1–2 µm in diameter, IKI–, CB–, tissues unchanged in KOH. ***Hymenium***. Cysitidia clavate to subclavate, colorless, thin-walled, occasionally sinuous in the basal position, smooth, 9–13.5 × 3.5–5 µm. Hyphidia arising from generative hyphae, rarely branched, colorless, thin-walled, 1.5–2.5 µm in diameter. Basidia subellipsoid to ovoid, longitudinally septate, two- to four-celled, 10.5–15 × 6.5–8.5 µm. Basidioles dominant, similar to basidia in shape, but slightly smaller. ***Basidiospores***. Allantoid to subcylindrical, slightly curved, colorless, thin-walled, smooth, IKI–, CB–, (6.5–)7.5–9.5(–10) × 4–5 µm, *L* = 8.63 µm, *W* = 4.54 µm, *Q* = 1.86–1.90 (*n* = 60/2).

##### Additional specimen examined (Paratype).

China • Yunnan Province, Dehong, Ruili City, Tongbiguan Provincial Nature Reserve, GPS coordinates 24°03'N, 97°42'E, altitude 1980 m asl., on the dead bamboo, leg. C.L. Zhao, 15 January 2025, CLZhao 44349 (SWFC 00044349).

#### Nigrochaete ellipsoidea

Taxon classificationFungiAuricularialesBasidiomycota

Q. Yuan & C.L. Zhao sp. nov.

0DD2BBD7-64D5-51ED-A477-059A202401E2

861885

Figs 7, 8, 9

##### Diagnosis.

It is characterized by its soft membranaceous basidiomata with slightly odontioid hymenial surface, a monomitic hyphal system with clamped generative hyphae, and ellipsoid to broadly ellipsoid basidiospores measuring 5–6 × 4–4.5 µm.

**Figure 7.**
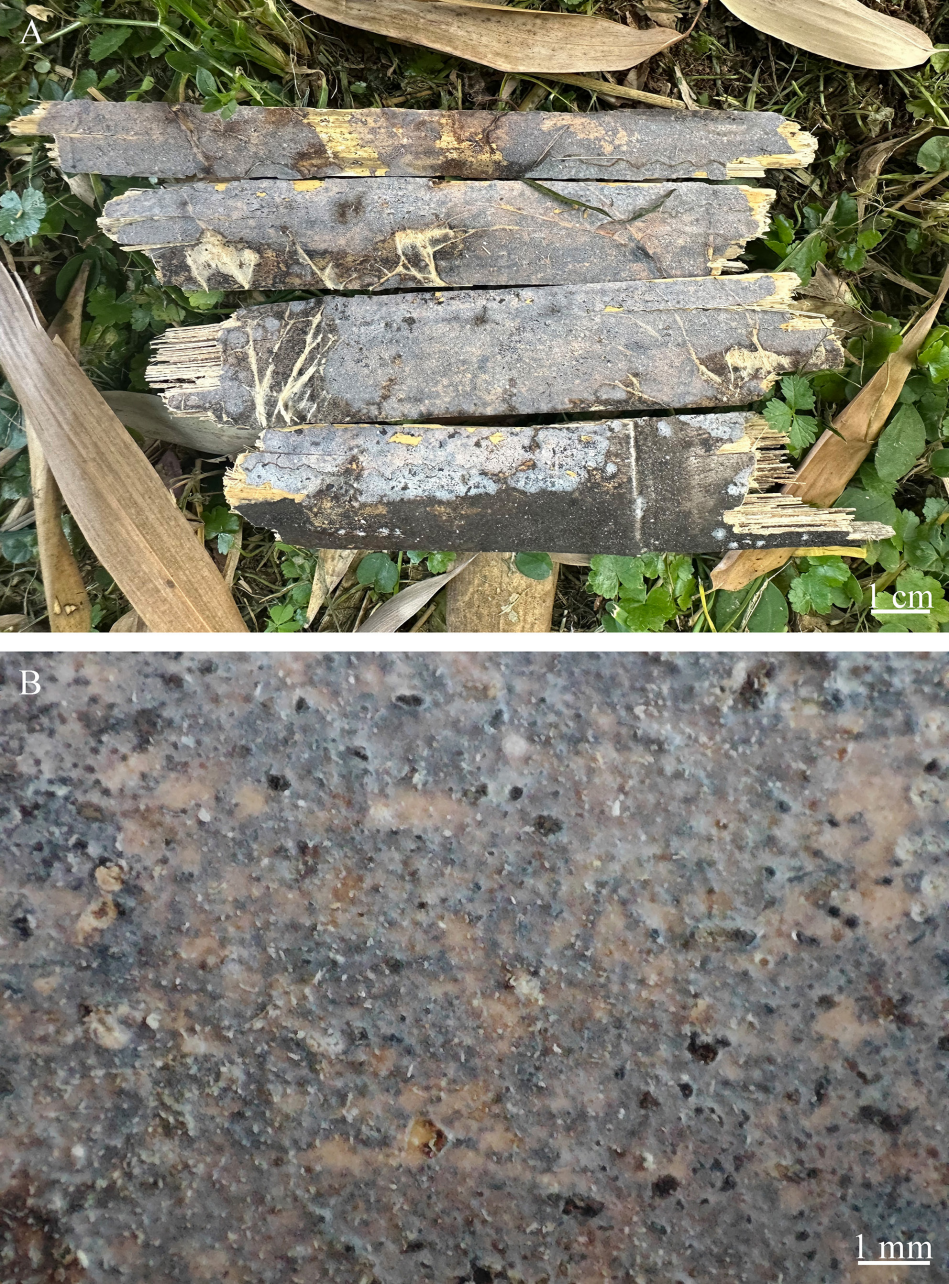
Basidiomata of *Nigrochaete ellipsoidea* in general and detailed views (CLZhao 45047, holotype).

**Figure 8.**
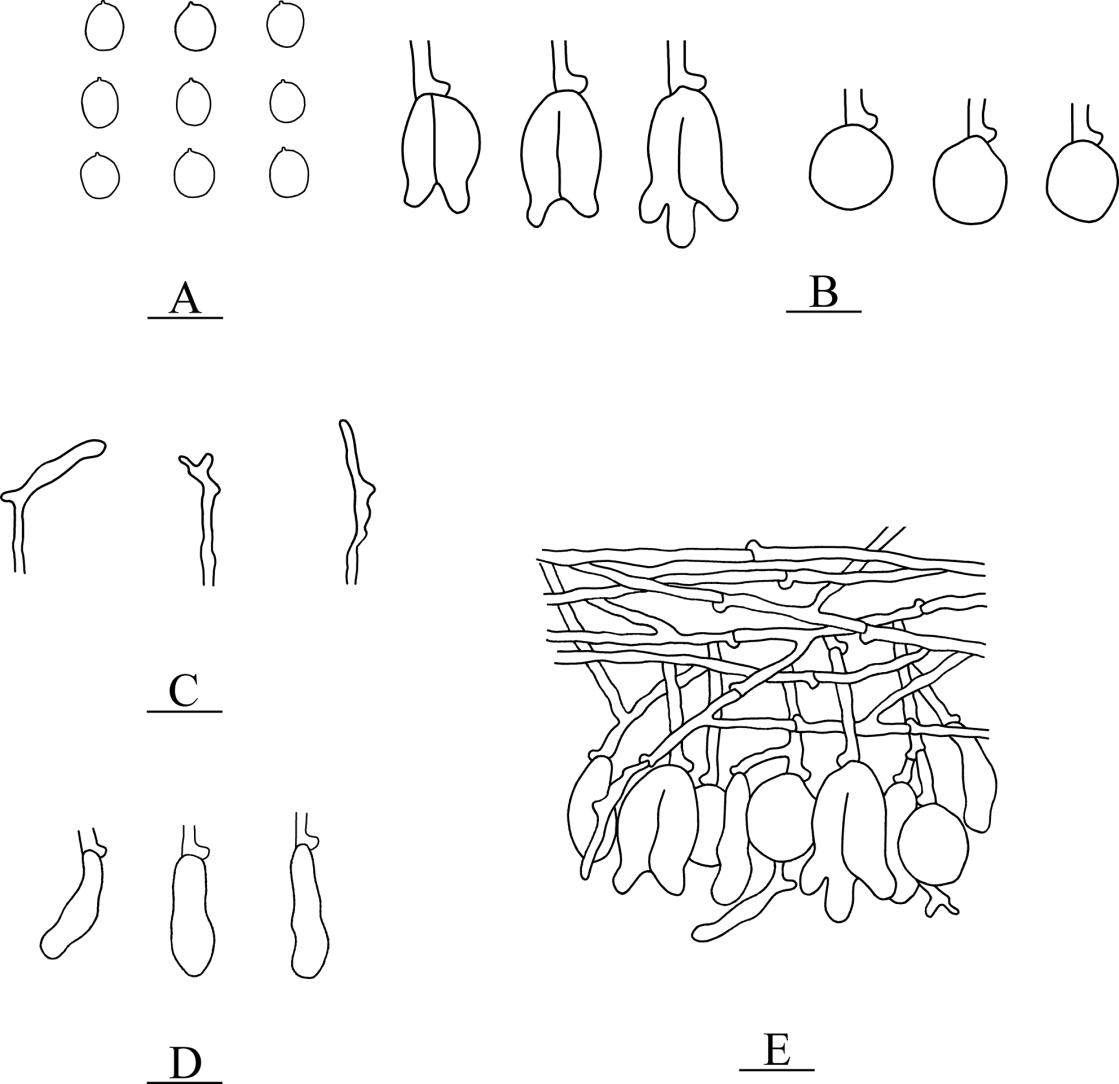
Microscopic structures of *Nigrochaete ellipsoidea* (CLZhao 45047, holotype). **A**. Basidiospores; **B**. Basidia and basidioles; **C**. Hyphidia; **D**. Cystidia; **E**. Part of the vertical section of the hymenium. Scale bars: 10 µm (**A–E**).

**Figure 9.**
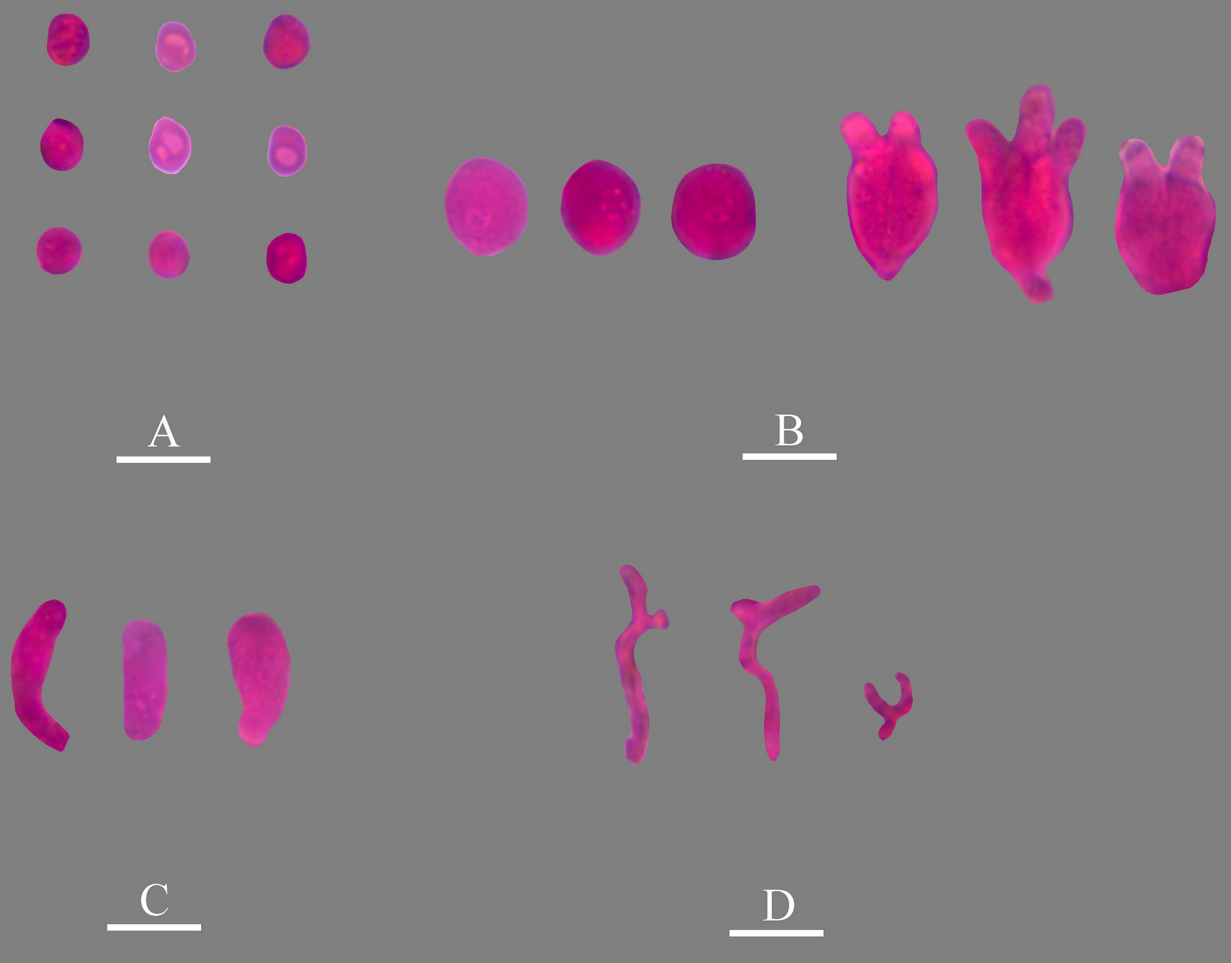
Sections of the hymenium of *Nigrochaete ellipsoidea* (CLZhao 45047, holotype). **A**. Basidiospores; **B**. Basidia and basidioles; **C**. Cystidia; **D**. Hyphidia. Scale bars: 10 µm (**A–D**); 10 × 100 oil.

##### Holotype.

China • Yunnan Province, Dehong, Ruili City, Tongbiguan Provincial Nature Reserve, GPS coordinates 24°01'N, 97°41'E, altitude 1920 m asl., on the dead bamboo, leg. C.L. Zhao, 16 January 2025, CLZhao 45047 (SWFC F00045047).

##### Etymology.

ellipsoidea (Lat.) refers to the type specimen having ellipsoid basidiospores.

##### Basidiomata.

Annual, resupinate, soft membranaceous, thin, closely adnate, without odor or taste when fresh, up to 12 cm long, 2.5 cm wide, and up to 50 µm thick. Hymenial surface odontioid, white when fresh, turning slightly cream upon drying. Sterile margin narrow, white, up to 1 mm.

##### Hyphal system.

Monomitic, generative hyphae with clamp connections, colorless, thin-walled, smooth, interwoven, 1.5–2 µm in diameter, IKI–, CB–, tissues unchanged in KOH. ***Hymenium***. Cysitidia subclavate, colorless, thin-walled, occasionally sinuous in the basal or middle position, smooth, 12.5–18 × 4–6 µm. Hyphidia arising from generative hyphae, rarely branched, colorless, thin-walled, 1.5–2.5 µm in diameter. Basidia subellipsoid to ovoid, longitudinally septate, two- to four-celled, 13.5–14.5 × 8–9.5 µm. Basidioles dominant, similar to basidia in shape, but slightly smaller. ***Basidiospores***. Ellipsoid to broadly ellipsoid, colorless, thin-walled, smooth, IKI–, CB–, 5–6 × 4–4.5(–5) µm, *L* = 5.39 µm, *W* = 4.29 µm, *Q* = 1.25 (*n* = 30/1).

#### Nigrochaete tenuis

Taxon classificationFungiAuricularialesBasidiomycota

Q. Yuan & C.L. Zhao, sp. nov .

F0BA6605-D293-5BB8-A426-2AED63B6D542

861886

Figs 10, 11, 12

##### Diagnosis.

It is characterized by its hard membranaceous basidiomata with smooth hymenial surface, a monomitic hyphal system with clamped generative hyphae, and broadly ellipsoid to globose basidiospores measuring 6.5–8 × 5.5–6.5 µm.

**Figure 10.**
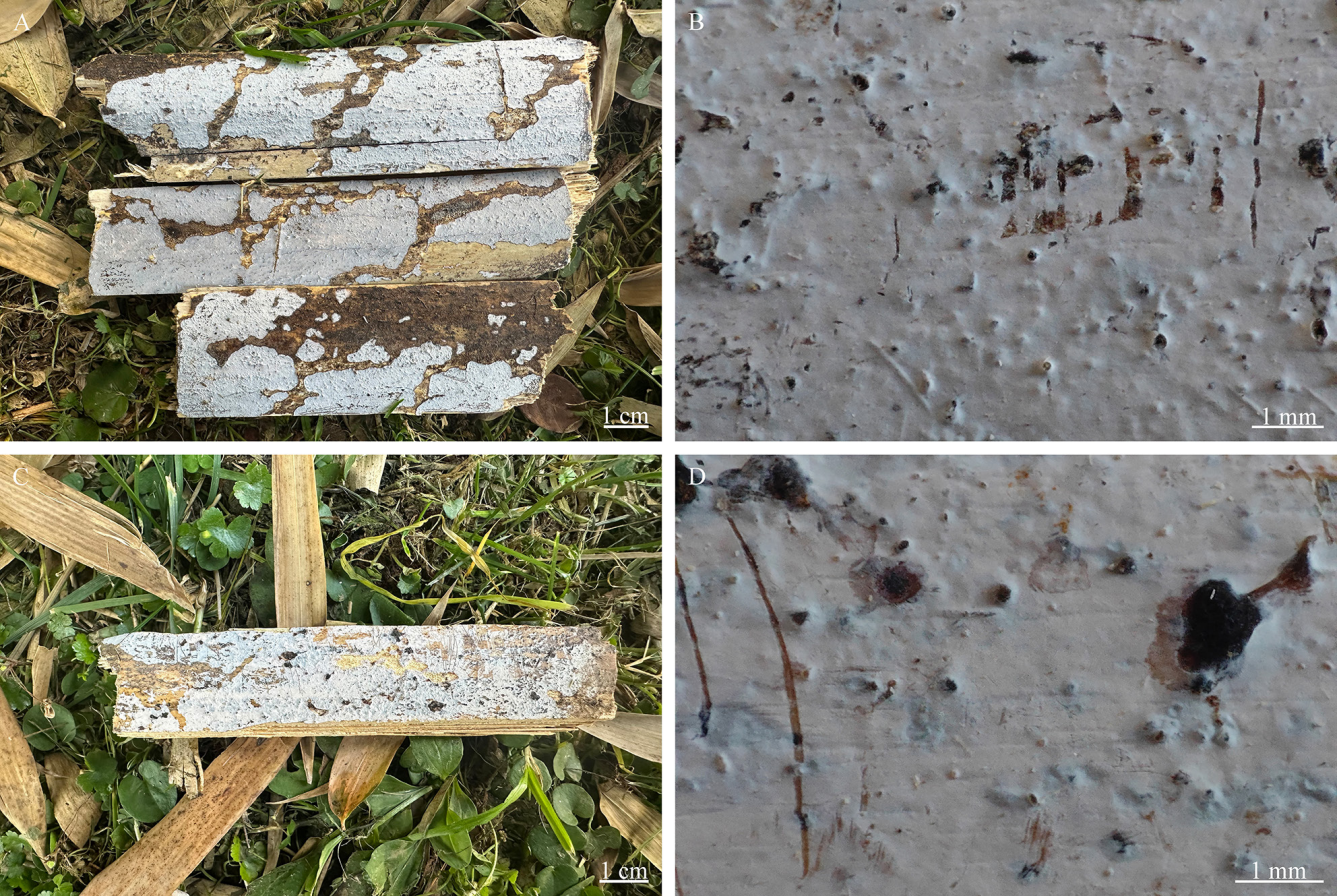
Basidiomata of *Nigrochaete tenuis* (CLZhao 44159, holotype). **A, B**. CLZhao 44159; **C, D**. CLZhao 44987.

**Figure 11.**
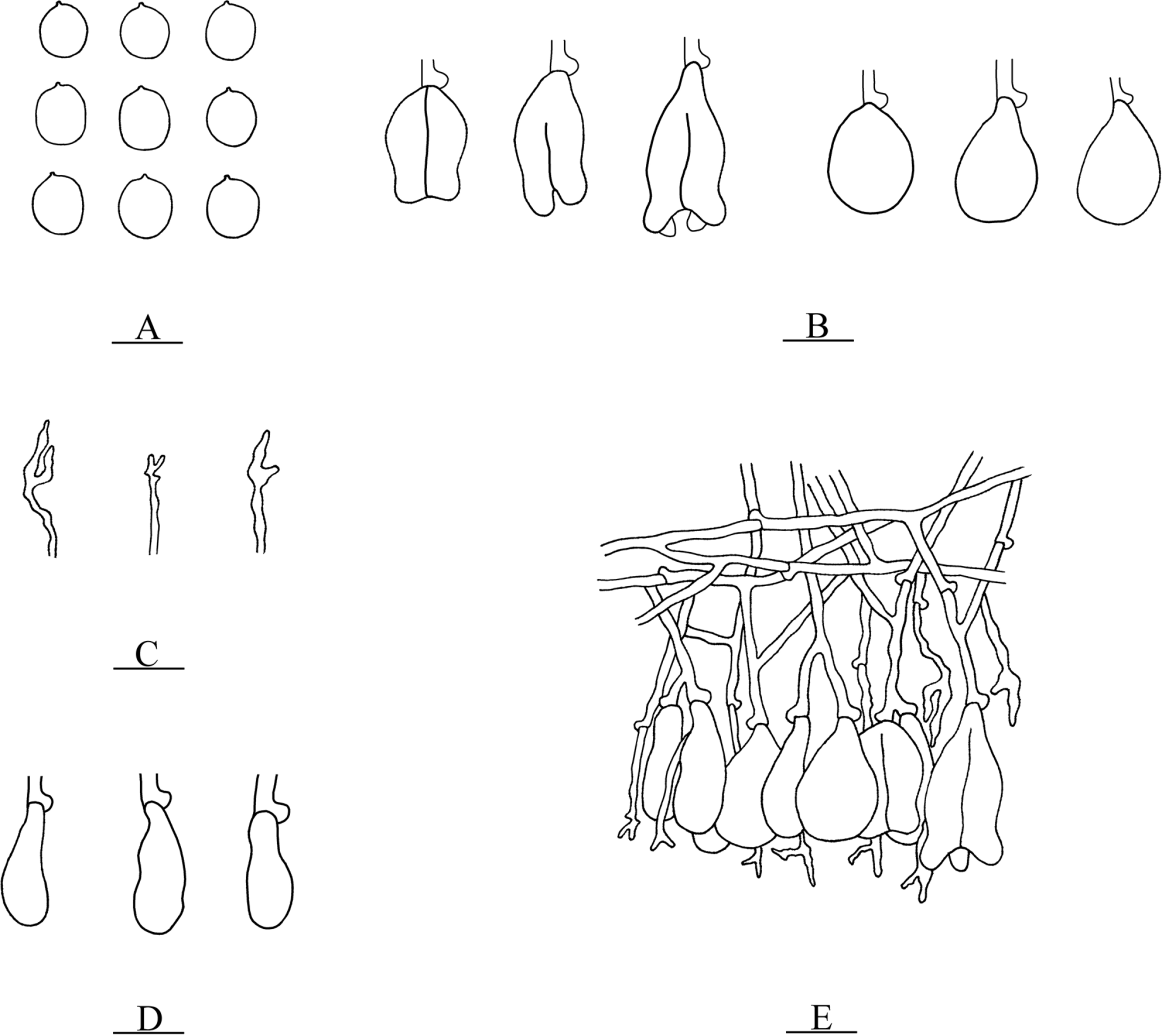
Microscopic structures of *Nigrochaete tenuis* (CLZhao 44159, holotype). **A**. Basidiospores; **B**. Basidia and basidioles; **C**. Hyphidia; **D**. Cystidia; **E**. Part of the vertical section of the hymenium. Scale bars: 10 µm (**A–E**).

**Figure 12.**
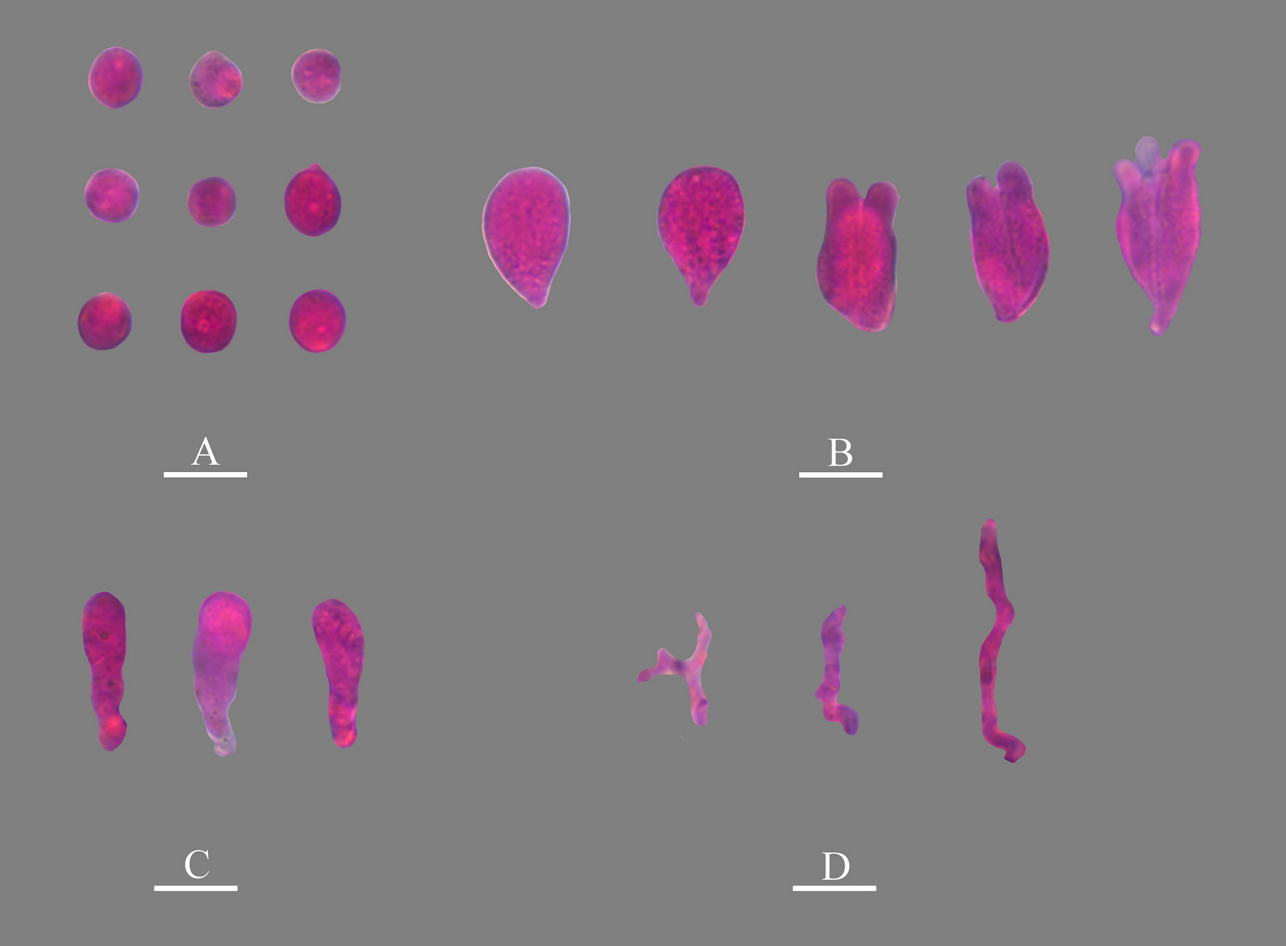
Sections of the hymenium of *Nigrochaete tenuis* (CLZhao 44159, holotype). **A**. Basidiospores; **B**. Basidia and basidioles; **C**. Cystidia; **D**. Hyphidia. Scale bars: 10 µm (**A–D**); 10 × 100 oil.

##### Holotype.

China • Yunnan Province, Dehong, Ruili City, Tongbiguan Provincial Nature Reserve, GPS coordinates 24°05'N, 97°15'E, altitude 2150 m asl., on the dead bamboo, leg. C.L. Zhao, 14 January 2025, CLZhao 44159 (SWFC 00044159).

##### Etymology.

tenuis (Lat.) refers to thin basidiomata of the type specimens.

##### Basidiomata.

Annual, resupinate, membranaceous, closely adnate, becoming hard membranaceous upon drying, without odor or taste when fresh, up to 11 cm long, 3 cm wide, and up to 100 µm thick. Hymenial surface smooth, white when fresh and drying. Sterile margin narrow, white, up to 1 mm.

##### Hyphal system.

Monomitic, generative hyphae with clamp connections, colorless, thin-walled, smooth, interwoven, 1.5–2 µm in diameter, IKI–, CB–, tissues unchanged in KOH. ***Hymenium***. Cysitidia subclavate, colorless, thin-walled, occasionally sinuous in the basal position, smooth, 15.5–17 × 4.5–6 µm. Hyphidia arising from generative hyphae, rarely branched, colorless, thin-walled, 1–3 µm in diameter. Basidia subellipsoid to ovoid, longitudinally septate, two- to four-celled, 12.5–18 × 8.5–10.5 µm. Basidioles dominant, similar to basidia in shape, but slightly smaller. ***Basidiospores***. Broadly ellipsoid to globose, colorless, thin-walled, smooth, IKI–, CB–, 6.5–8(–8.5) × 5.5–6.5 µm, *L* = 7.25 µm, *W* = 6.19 µm, *Q* = 1.17–1.19 (*n* = 60/2).

##### Additional specimen examined (Paratype).

China • Yunnan Province, Dehong, Ruili City, Tongbiguan Provincial Nature Reserve, GPS coordinates 24°01'N, 97°41'E, altitude 1920 m asl., on the dead bamboo, leg. C.L. Zhao, 16 January 2025, CLZhao 44987 (SWFC 00044987).

## Discussion

In the present study, three new species, *Nigrochaete albobadia*, *N. ellipsoidea*, and *N. tenuis*, are described based on phylogenetic analyses and morphological characteristics.

The corticioid species of *Auriculariales* are traditionally placed in the genera *Eichleriella*, *Exidiopsis*, and *Heterochaete* primarily based on morphological features (Liu et al. 2022). However, recent taxonomic revisions supported by molecular evidence have led to the establishment of several new corticioid genera, including *Adustochaete*, *Alloexidiopsis*, *Amphistereum*, *Crystallodon*, *Proterochaete*, and *Sclerotrema*, as well as the reinstatement of previously recognized genera, such as *Hirneolina*, *Heteroradulum*, and *Tremellochaete* (Malysheva and Spirin 2017; Alvarenga et al. 2019; Alvarenga and Gibertoni 2021). Multilocus phylogenetic analyses with broader sampling across various morphological groups in *Auriculariales* have demonstrated that achieving a more natural and stable classification of the order is possible, as in other lineages of *Agaricomycetes* (Wang and Thorn 2021).

Morphologically, *Nigrochaete albobadia*, *N. ellipsoidea*, and *N. tenuis* resemble *N. bambusicola* by having subellipsoid to ovoid basidia (Dong et al. 2025a). However, *N. bambusicola* can be separated from *N. albobadia* by its coriaceous basidiomata with a grayish-black to black, grandinoid hymenial surface and longer basidiospores (11.3–13.2 × 4.5–5 µm vs. 7.5–9.5 × 4–5 µm) (Dong et al. 2025a). *N. bambusicola* is distinct from *N. ellipsoidea* by its grayish-black to black hymenial surface, longer basidia (16–18.5 × 7.5–9.5 µm vs. 13.5–14.5 × 8–9.5 µm), and longer basidiospores (11.3–13.2 × 4.5–5 µm vs. 5–6 × 4–4.5 µm) (Dong et al. 2025a). *N. bambusicola* is separated from *N. tenuis* by the coriaceous basidiomata with a grayish-black to black, grandinoid hymenial surface and longer basidiospores (11.3–13.2 × 4.5–5 µm vs. 6.5–8 × 5.5–6.5 µm) (Dong et al. 2025a). A morphological comparison between the three new *Nigrochaete* species and *N. bambusicola* is presented in Table 4.

**Table 4.** A morphological comparison between three new *Nigrochaete* species and one similar species in the genus *Nigrochaete*. Newly introduced taxa are indicated in bold black font.

Species name	Basidiomata/Hymenial surface	Hyphae	Cystidia	Basidia	Basidiospores	References
***Nigrochaete albobadia***	Membranaceous; smooth, white	Thin-walled, branched	Clavate to subclavate; 9–13.5 × 3.5–5 µm	Subellipsoid to ovoid, two- to four-celled; 10.5–15 × 6.5–8.5 µm	Allantoid to subcylindrical, slightly curved; 7.5–9.5 × 4–5 µm	**Present study**
*Nigrochaete bambusicola*	Coriaceous; grandinoid, grayish‐black to black	Thin-walled, branched	Subclavate to subcylindrical; 17–25 × 6–10 µm	Subellipsoid to ovoid, two- to four-celled; 16–18.5 × 7.5–9.5 µm	Allantoid, slightly curved; 11.3–13.2 × 4.5–5 µm	Dong et al. (2025a)
***Nigrochaete ellipsoidea***	Soft membranaceous; odontioid, slightly cream	Thin-walled, branched	Subclavate; 12.5–18 × 4–6 µm	Subellipsoid to ovoid, two- to four-celled; 13.5–14.5 × 8–9.5 µm	Ellipsoid to broadly ellipsoid; 5–6 × 4–4.5 µm	**Present study**
***Nigrochaete tenuis***	Hard membranaceous; smooth, white	Thin-walled, branched	Subclavate; 15.5–17 × 4.5–6 µm	Subellipsoid to ovoid, two- to four-celled; 12.5–18 × 8.5–10.5 µm	Broadly ellipsoid to globose; 6.5–8 × 5.5–6.5 µm	**Present study**

Phylogenetically, based on the combined ITS+nrLSU sequence data (Fig. 1), the three new species were nested within the genus *Nigrochaete* within *Auriculariales*. Based on the ITS+nrLSU+*TEF*1 topology tree (Fig. 2), *N. albobadia* was retrieved as sister to *N. tenuis*, whereas *N. ellipsoidea* constituted an independent lineage closely related to *N. bambusicola*. However, *N. tenuis* differs from *N. albobadia* by its hard membranaceous basidiomata, wider basidia (12.5–18 × 8.5–10.5 µm vs. 10.5–15 × 6.5–8.5 µm), and wider basidiospores (6.5–8 × 5.5–6.5 µm vs. 7.5–9.5 × 4–5 µm). The species *N. bambusicola* can be distinguished from *N. ellipsoidea* by its coriaceous basidiomata with a grayish-black to black hymenial surface, longer basidia (16–18.5 × 7.5–9.5 µm vs. 13.5–14.5 × 8–9.5 µm), and longer basidiospores (11.3–13.2 × 4.5–5 µm vs. 5–6 × 4–4.5 µm) (Dong et al. 2025a). The taxon *N. albobadia* differs from *N. tenuis* by its membranaceous basidiomata, narrower basidia (10.5–15 × 6.5–8.5 µm vs. 12.5–18 × 8.5–10.5 µm), and narrower basidiospores (7.5–9.5 × 4–5 µm vs. 6.5–8 × 5.5–6.5 µm).

From an ecological perspective, all currently known species of *Nigrochaete* were collected from dead bamboo and are so far restricted to Dehong Dai and Jingpo Autonomous Prefecture, Yunnan Province, China (Dong et al. 2025a). This region is characterized by a South Asian tropical monsoon climate. Therefore, based on the specimens collected to date, this genus is hypothesized to be endemic to this specific climatic zone and to act as a host-specific saprobe on bamboo. This pattern suggests possible ecological specialization and indicates that the genus’s diversity remains underexplored. Additional species of *Nigrochaete* are likely to be discovered as sampling increases across different geographic regions and host substrates.

Fungi represent one of the most diverse groups of organisms on Earth and play a crucial role in ecosystem processes and functions (Case et al. 2025; Dai et al. 2025; He et al. 2025; Hongsanan et al. 2025; Xu et al. 2025; Yang et al. 2025). Advances in DNA sequencing techniques have revolutionized studies of fungal taxonomy and diversity, with approximately 155,000 species formally described to date (Niskanen et al. 2023). Wood-inhabiting fungi are an extensively studied group of *Basidiomycota*, which includes a number of poroid, smooth, grandinoid, odontioid, and hydnoid basidiomata in China (Wang et al. 2025). In the past several years, many corticioid species have been reported and described in the order *Auriculariales* (Wells and Bandoni 2001; Miettinen et al. 2012; Hibbett et al. 2014; Malysheva and Spirin 2017; Alvarenga et al. 2019; Spirin et al. 2019a, 2019b; Tohtirjap et al. 2023; Dong et al. 2025a; Spirin et al. 2025). Nevertheless, the corticioid diversity of *Auriculariales* remains insufficiently known, particularly in subtropical and tropical regions of China.

In conclusion, the discovery and description of *Nigrochaete albobadia*, *N. ellipsoidea*, and *N. tenuis* further enrich our understanding of species diversity within *Auriculariales*. Continued field surveys combined with integrative morphological and molecular approaches will undoubtedly reveal additional undescribed corticioid taxa, and future collections may also uncover species of *Nigrochaete* occurring on angiosperms or other host substrates.

### Key to the known species of *Nigrochaete* worldwide

**Table:** 

1	Hymenial surface smooth	**2**
–	Hymenial surface grandinoid or odontioid	**3**
2	Basidiomata hard membranaceous, basidiospores > 5 µm wide	***Nigrochaete tenuis***
–	Basidiomata membranaceous, basidiospores < 5 µm wide	***Nigrochaete albobadia***
3	Basidiomata coriaceous, basidiospores > 4.5 µm wide	***Nigrochaete bambusicola***
–	Basidiomata soft membranaceous, basidiospores < 4.5 µm wide	***Nigrochaete ellipsoidea***

## Supplementary Material

XML Treatment for Nigrochaete albobadia

XML Treatment for Nigrochaete ellipsoidea

XML Treatment for Nigrochaete tenuis

## Additional information

### Artificial Intelligence (AI) use

The authors accept full responsibility for the content of the manuscript, including the disclosure of any use of AI.

No AI tools were used in the preparation of this manuscript.

### Author ORCIDs

Q. Yuan https://orcid.org/0000-0002-6732-1656

Q. Chen https://orcid.org/0009-0009-6562-1282

S. You https://orcid.org/0009-0005-3948-6217

X. Liu https://orcid.org/0000-0003-0100-2094

C. Zhao https://orcid.org/0000-0002-8668-1075

### Data availability

All of the data that support the findings of this study are available in the main text.

## References

[B1] Alvarenga RLM, Gibertoni TB (2021) *Crystallodon* Alvarenga gen. nov., a new genus of the *Auriculariales* from the Neotropics. Cryptogamie. Mycologie 42(2): 17–24. 10.5252/cryptogamie-mycologie2021v42a2

[B2] Alvarenga RLM, Spirin V, Malysheva V, Gibertoni TB, Larsson KH (2019) Two new genera and six other novelties in *Heterochaete**sensu lato* (*Auriculariales*, *Basidiomycota*). Botany 97(8): 439–451. 10.1139/cjb-2019-0046

[B3] Case NT, Gurr SJ, Fisher MC, Blehert DS, Boone C, Casadevall A, Chowdhary A, Cuomo CA, Currie CR, Denning DW, Ene IV, Fritz-Laylin LK, Gerstein AC, Gow NAR, Gusa A, Iliev ID, James TY, Jin HL, Kahmann R, Klein BS, Kronstad JW, Ost KS, Peay KG, Shapiro RS, Sheppard DC, Shlezinger N, Stajich JE, Stukenbrock EH, Taylor JW, Wright GD, Cowen LE, Heitman J, Segre JA (2025) Fungal impacts on Earth’s ecosystems. Nature 638: 49–57. 10.1038/s41586-024-08419-4 PMC11970531 39910383

[B4] Cui YY, Fan XP, Guo LJ, Yang ZL (2024) New elements of funga from southwestern China: species of *Tremellodendropsis* and *Guepinia*. Mycosystema 43: 230266. 10.13346/j.mycosystema.230266

[B5] Dai YF, Yuan Q, Yang X, Liu R, Liu DF, Yuan HS, Zhao CL (2025) Morphological characteristics and phylogenetic analyses reveal five new species of *Hymenochaetales* (*Agaricomycetes*, *Basidiomycota*) from southwestern China. MycoKeys 114: 133–175. 10.3897/mycokeys.114.143851 PMC11883501 40051985

[B6] Deng YL, Chen M, Zhang SC, Wang KS, Liu WT, Qiu YH, Dou YT, Liu XF, Wijesinghe ASN, Zhou HM, Jabeen S, Zhao CL (2026) Notes, taxonomy, and phylogeny of wood-inhabiting fungi in *Russulales*. Mycosphere 17: e003.

[B7] Dissanayake AJ, Bhunjun CS, Maharachchikumbura SS, Liu JK (2020) Applied aspects of methods to infer phylogenetic relationships amongst fungi. Mycosphere 11: 2652–76. 10.5943/mycosphere/11/1/18

[B8] Dong JH, Zhu YG, Qian CB, Zhao CL (2024) Taxonomy and phylogeny of *Auriculariales* (*Agaricomycetes*, *Basidiomycota*) with descriptions of four new species from south-western China. MycoKeys 108: 115–146. 10.3897/mycokeys.108.128659 PMC11380053 39246551

[B9] Dong JH, Zhang JL, He SY, Chen ML, Zhao CL (2025a) A new corticioid genus of *Auriculariales*—*Nigrochaete*, collected from Yunnan China. Journal of Fungal Research 23: 190–201. 10.13341/j.jfr.2024.1826

[B10] Dong JH, Chen ML, Chen M, Li Q, Zhu YJ, Zhang XC, Zhou CQ, Li W, Muhammad A, Zhou HM, Jabeen S, Zhao CL (2025b) Notes, outline, taxonomy and phylogeny of wood-inhabiting *Agaricales*. Mycosphere 16: 2599–2711. 10.5943/mycosphere/16/1/16

[B11] Dong JH, Xu Y, Jiang QQ, Hosen MI, Zhao CL (2025c) A new genus and two new species of *Auriculariales (Basidiomycota)* from southwest China, evidenced by morphological characteristics and phylogenetic analyses. Mycological Progress 24: 1–17. 10.21203/rs.3.rs-5169056/v1

[B12] Dong JH, Li Q, Su JQ, Zhao CL (2025d) *Punctochaete murina* gen. et sp. nov. (*Agaricomycetes*, *Basidiomycota*) from southwestern China. European Journal of Taxonomy 981: 96–113. 10.5852/ejt.2025.981.2821

[B13] Glez-Peña D, Gómez-Blanco D, Reboiro-Jato M, Fdez-Riverola F, Posada D (2010) ALTER: program-oriented conversion of DNA and protein alignments. Nucleic Acids Research 38: 14–18. 10.1093/nar/gkq321 PMC2896128 20439312

[B14] He MQ, Cao B, Liu F, Boekhout T, Denchev TT, Schoutteten N, Denchev CM, Kemler M, Gorjón SP, Begerow D, Valenzuela R, Davoodian N, Niskanen T, Vizzini A, Redhead SA, Ramírez-Cruz V, Papp V, Dudka VA, Dutta AK, García-Sandoval R, Liu XZ, Kijpornyong pan T, Savchenko A, Tedersoo L, Theelen B, Trierveiler-Pereira L, Wu F, Zamora JC, Zeng XY, Zhou LW, Liu SL, Ghobad-Nejhad M, Giachini AJ, Li GJ, Kakishima M, Olariaga I, Haelewaters D, Sulistyo B, Sugiyama J, Svantesson S, Yurkov A, Alvarado P, Antonín V, da Silva AF, Druzhinina I, Gibertoni TB, Guzmán-Dávalos L, Justo A, Karunarathna SC, Galappaththi MCA, Toome-Heller M, Hosoya T, Liimatainen K, Márquez R, Mešić A, Moncalvo JM, Nagy LG, Varga T, Orihara T, Raymundo T, Salcedo I, Silva-Filho AGS, Tkalčec Z, Wartchow F, Zhao CL, Bau T, Cabarroi-Hernández1 M, Cortés-Pérez A, De cock C, Lange RD, Weiss M, Menolli Jr N, Nilsson RH, Fan YG, Verbeken A, Gaforov Y, Meiras-Ottoni A, Mendes-Alvarenga RL, Zeng NK, Wu Q, Hyde KD, Kirk PM, Zhao RL (2024) Phylogenomics, divergence times and notes of orders in *Basidiomycota*. Fungal Diversity 99: 105–367. 10.1007/s13225-024-00535-w

[B15] He SY, Wang L, Shen KZ, Zhou HM (2025) Morphological characteristics and phylogenetic analyses revealed four new species (*Basidiomycota*) in the Yunnan-Guizhou Plateau, China. MycoKeys 113: 237–262. 10.3897/mycokeys.113.140932 PMC11840434 39980723

[B16] Hibbett DS, Binder M, Bischoff JF, Blackwell M, Cannon PF, Eriksson OE, Huhndorf S, James T, Kirk PM, Lücking R, Thorsten Lumbsch H, Lutzoni F, Matheny PB, McLaughlin DJ, Powell MJ, Redhead S, Schoch CL, Spatafora JW, Stalpers JA, Vilgalys R, Aime MC, Aptroot A, Bauer R, Begerow D, Benny GL, Castlebury LA, Crous PW, Dai YC, Gams W, Geiser DM, Griffith GW, Gueidan C, Hawksworth DL, Hestmark G, Hosaka K, Humber RA, Hyde KD, Ironside JE, Kõljalg U, Kurtzman CP, Larsson KH, Lichtwardt R, Longcore J, Miadlikowska J, Miller A, Moncalvo JM, Mozley-Standridge S, Oberwinkler F, Par masto E, Reeb V, Rogers JD, Roux C, Ryvarden L, Sampaio JP, Schüssler A, Sugiyama J, Thorn RG, Tibell L, Untereiner WA, Walker C, Wang Z, Weir A, Weiss M, White MM, Winka K, Yao YJ, Zhang N (2007) A higher-level phylogenetic classification of the *Fungi*. Mycological Research 111(5): 509–547. 10.1016/j.mycres.2007.03.004 17572334

[B17] Hibbett DS, Bauer R, Binder M, Giachini AJ, Hosaka K, Justo A, Larsson E, Larsson KH, Lawrey JD, Miettinen O, Nagy LG, Nilsson RH, Weiss M, Thorn RG (2014) 14 *Agaricomycetes*. In: McLaughlin D, Spatafora J (Eds) Systematics and Evolution. The Mycota, vol 7A. Springer, Berlin, Heidelberg, 373–430. 10.1007/978-3-642-55318-9_14

[B18] Hongsanan S, Khuna S, Manawasinghe IS, Tibpromma S, Chethana KWT, Xie N, Bagacay JFE, Calabon MS, Chen C, Doilom M, Du HY, Gafforov Y, Huang SK, Li JX, Luangharn T, Luo ZL, Opiña LAD, Pem D, Sadaba RB, Singh R, Tan Q, Tang SM, Wang WP, Wen TC, Xia G, Zhao Q, Bhunjun CS, Cao B, Chen YP, de Silva NI, Dai DQ, Dong W, Du TY, Ferreira-Sá AS, Gao Y, Gui H, Han LS, Han MY, Han XX, Jayawardena RS, Khyaju S, Kumar S, Lei L, Leonardo-Silva L, Li H, Li YX, Liao CF, Liu JW, Liu XF, Lu L, Lu WH, Luo M, Maharachchikumbura SSN, Meng QF, Mi LX, Norphanphoun C, Peng XC, Su HL, Tennakoon DS, Thiyagaraja V, Tun ZL, Wijayawardene NN, Xavier-Santos S, Xiong YR, Xu RF, Yadav S, Yang T, Yang YH, Yarasheva M, Zeng XY, Zhang H, Zhang GQ, Zhang X, Zhao HJ, Zhao RL, Zheng DG, Wanasinghe DN, Karunarathna SC (2025) Mycosphere Notes 521–571: A special edition of fungal biodiversity to celebrate Kevin D. Hyde’s 70^th^ birthday and his exceptional contributions to mycology. Mycosphere 16(2): 1–178. 10.5943/mycosphere/16/2/1

[B19] Jeewon R, Hyde KD (2016) Establishing species boundaries and new taxa among fungi: recommendations to resolve taxonomic ambiguities. Mycosphere 7(11): 1669–1677. 10.5943/mycosphere/7/11/4

[B20] Kalyaanamoorthy S, Minh BQ, Wong TKF, Haeseler AV, Jermiin LS (2017) ModelFinder: Fast model selection for accurate phylogenetic estimates. Nature Methods 14: 587–589. 10.1038/nmeth.4285 PMC5453245 28481363

[B21] Katoh K, Rozewicki J, Yamada KD (2019) MAFFT online service: multiple sequence alignment, interactive sequence choice and visualization. Briefings in Bioinformatics 20: 1160–1166. 10.1093/bib/bbx108 PMC6781576 28968734

[B22] Kirschner R, Chen CJ (2004) *Helicomyxa everhartioides*, a new helicosporous sporodochial hyphomycete from Taiwan with relationships to the *Hyaloriaceae* (*Auriculariales*, *Basidiomycota*). Studies in Mycology 50: 337–342.

[B23] Kirschner R, Yang ZL, Zhao Q, Feng B (2010) *Ovipoculum album*, a new anamorph with gelatinous cupulate bulbilliferous conidiomata from China and with affinities to the *Auriculariales (Basidiomycota)*. Fungal Diversity 43: 55–65. 10.1007/s13225-010-0038-0

[B24] Kirschner R, Lee IS, Piepenbring M (2012) A new pycnidial fungus with clamped hyphae from Central America. Mycological Progress 11: 561–568. 10.1007/s11557-011-0771-0

[B25] Larsson A (2014) AliView: a fast and lightweight alignment viewer and editor for large data sets. Bioinformatics 30: 3276–3278. 10.1093/bioinformatics/btu531 PMC4221126 25095880

[B26] Larsson KH (2007) Re-thinking the classification of corticioid fungi. Mycological Research 111: 1040–1063. 10.1016/j.mycres.2007.08.001 17981020

[B27] Li Y, Nie T, Nakasone KK, Li HJ, He SH (2023) Taxonomy and phylogeny of corticioid fungi in *Auriculariaceae* (*Auriculariales*, *Basidiomycota*): A new genus, five new species and four new combinations. Journal of Fungi 9: 318. 10.3390/jof9030318 PMC10056916 36983486

[B28] Liu SL, Shen ZQ, Li QZ, Liu XY, Zhou LW (2022) *Alloexidiopsis* gen. nov., a revision of generic delimitation in *Auriculariales (Basidiomycota)*. Frontiers in Microbiology 13: 894641. 10.3389/fmicb.2022.894641 PMC9315202 35903469

[B29] Lutzoni F, Kauff F, Cox CJ, McLaughlin D, Celio G, Dentinger B, Padamsee M, Hibbett D, James TY, Baloch E, Grube M, Reeb V, Hofstetter V, Schoch C, Arnold AE, Miadlikowska J, Spatafora J, Johnson D, Hambleton S, Crockett M, Shoemaker R, Sung GH, Lücking R, Lumbsch T, O’Donnell K, Binder M, Diederich P, Ertz D, Gueidan C, Hansen K, Harris RC, Hosaka K, Lim YW, Matheny B, Nishida H, Pfister D, Rogers J, Rossman A, Schmitt I, Sipman H, Stone J, Sugiyama J, Yahr R, Vilgalys R (2004) Assembling the fungal tree of life: progress, classification, and evolution of subcellular traits. American Journal of Botany 91(10): 1446–80. 10.3732/ajb.91.10.1446 21652303

[B30] Malysheva V, Spirin V (2017) Taxonomy and phylogeny of the *Auriculariales* (*Agaricomycetes*, *Basidiomycota*) with stereoid basidiocarps. Fungal Biology 121(8): 689–715. 10.1016/j.funbio.2017.05.001 28705397

[B31] Malysheva V, Spirin V, Miettinen O, Motato-Vásquez V, Hernawati JSSS, Larsson KH (2018) Revision of *Protohydnum* (*Auriculariales*, *Basidiomycota*). Mycological Progress 17(7): 805–814. 10.1007/s11557-018-1393-6

[B32] Miettinen O, Spirin V, Niemelä T (2012) Notes on the genus *Aporpium* (*Auriculariales*, *Basidiomycota*), with a new species from temperate Europe. Annales Botanici Fennici 49(5–6): 359–368. 10.5735/085.049.0607

[B33] Miller MA, Pfeiffer W, Schwartz T (2012) The CIPRES science gateway. In: Miller MA, Pfeiffer W, Schwartz T (Eds) Proceedings of the 1^st^ Conference of the Extreme Science and Engineering Discovery Environment: Bridging from the Extreme to the Campus and Beyond, Chicago, IL, 39 pp. 10.1145/2335755.2335836

[B34] Niskanen T, Lücking R, Dahlberg A, Gaya E, Suz LM, Mikryukov V, Liimatainen K, Druzhinina I, Westrip JRS, Mueller GM, Martins-Cunha K, Kirk P, Tedersoo L, Antonelli A (2023) Pushing the frontiers of biodiversity research: Unveiling the global diversity, distribution, and conservation of fungi. Annual Review of Environment and Resources 48: 149–176. 10.1146/annurev-environ-112621-090937

[B35] Petersen JH (1996) Farvekort. In: Petersen JH (Ed.) The Danish Mycological Society’s Colour-Chart, Foreningen til Svampekundskabens Fremme: Greve, Germany, 6 pp.

[B36] Rathnayaka AR, Tennakoon DS, Jones GE, Wanasinghe DN, Bhat DJ, Priyashantha AH, Stephenson SL, Tibpromma S, Karunarathna SC (2025) Significance of precise documentation of hosts and geospatial data of fungal collections, with an emphasis on plant-associated fungi. New Zealand Journal of Botany 63: 462–489. 10.1080/0028825X.2024.2381734

[B37] Rehner SA, Buckley E (2005) A *Beauveria* phylogeny inferred from nuclear ITS and EF1-α sequences evidence for cryptic diversification and links to *Cordyceps* teleomorphs. Mycologia 97: 84–98. 10.1080/15572536.2006.11832842 16389960

[B38] Ronquist F, Teslenko M, van der Mark P, Ayres DL, Darling A, Hohna S, Larget B, Liu L, Suchard MA, Huelsenbeck JP (2012) MrBayes 3.2: Efficient Bayesian phylogenetic inference and model choice across a large model space. Systematic Biology 61(3): 539–542. 10.1093/sysbio/sys029 PMC3329765 22357727

[B39] Schoutteten N, Yurkov A, Spirin V, Savchenko A, Aime MC, Begerow D, Verbeken A (2024) Examination of mycoparasites reveals a new type of host-parasite interface and rearranges the taxonomy of *Occultifur* and *Microsporomyces* (*Cystobasidiomycetes*, *Basidiomycota*). Studies in Mycology 109: 451–486. 10.3114/sim.2024.109.07 PMC11663426 40881650

[B40] Sotome K, Maekawa N, Nakagiri A, Lee SS, Hattori T (2014) Taxonomic study of Asian species of poroid *Auriculariales*. Mycological Progress 13: 987–997. 10.1007/s11557-014-0984-0

[B41] Spirin V, Malysheva V, Larsson KH (2018) On some forgotten of *Exidia* and *Myxarium* (*Auriculariales*, *Basidiomycota*). Nordic Journal of Botany 36: e01601. 10.1111/njb.01601

[B42] Spirin V, Malysheva V, Roberts P, Trichies G, Savchenko A, Larsson KH (2019a) A convolute diversity of the *Auriculariales* (*Agaricomycetes*, *Basidiomycota*) with sphaeropedunculate basidia. Nordic Journal of Botany 2019(7): e02394. 10.1111/njb.02394

[B43] Spirin V, Malysheva V, Miettinen O, Vlasák J, Alvarenga RLM, Gibertoni TB, Ryvarden L, Larsson KH (2019b) On *Protomerulius* and *Heterochaetella* (*Auriculariales*, *Basidiomycota*). Mycological Progress 18(9): 1079–1099. 10.1007/s11557-019-01507-0

[B44] Spirin V, Malysheva V, Haelewaters D, Larsson KH (2019c) Studies in the *Stypella vermiformis* group (*Auriculariales*, *Basidiomycota*). Antonie van Leeuwenhoek 112: 753–764. 10.1007/s10482-018-01209-9 PMC6456474 30535961

[B45] Spirin V, Malysheva V, Alvarenga RLM, Kotiranta H, Larsson KH (2020) Studies in *Basidiodendron eyrei* and similar-looking taxa (*Auriculariales*, *Basidiomycota*). Botany 98: 623–638. 10.1139/cjb-2020-0045

[B46] Spirin V, Malysheva V, Schoutteten N, Viner I, Miettinen O, Nordén J, Ryvarden L, Kotiranta H, Verbeken A, Weiß M, Larsson KH (2021) Studies in the *Basidiodendron caesiocinereum* complex (*Auriculariales*, *Basidiomycota*). Mycological Progress 20: 1275–1296. 10.1007/s11557-021-01724-6

[B47] Spirin V, Malysheva V, Viner I, Alvarenga RLM, Grebenc T, Gruhn G, Savchenko A, Grootmyers D, Ryvarden L, Vlasák J, Larsson KH, Nilsson RH (2025) Additions to the taxonomy of the *Auriculariales (Basidiomycota)* with pedunculate basidia. MycoKeys 120: 339–392. 10.3897/mycokeys.120.155492 PMC12381581 40881510

[B48] Tedersoo L, Bahram M, Põlme S, Kõljalg U, Yorou NS, Wijesundera R, Villarreal Ruiz L, Vasco-Palacios AM, Thu PQ, Suija A, Smith ME, Sharp C, Saluveer E, Saitta A, Rosas M, Riit T, Ratkowsky D, Pritsch K, Põldmaa K, Piepenbring M, Phosri C, Peterson M, Parts K, Pärtel K, Otsing E, Nouhra E, Njouonkou AL, Nilsson RH, Morgado LN, Mayor J, May TW, Majuakim L, Lodge DJ, Lee SS, Larsson KH, Kohout P, Hosaka K, Hiiesalu I, Henkel TW, Harend H, Guo LD, Greslebin A, Grelet G, Geml J, Gates G, Dunstan W, Dunk C, Drenkhan R, Dearnaley J, De Kesel A, Dang T, Chen X, Buegger F, Brearley FQ, Bonito G, Anslan S, Abell S, Abarenkov K (2014) Fungal biogeography. Global diversity and geography of soil fungi. Science 346(6213): 1256688. 10.1126/science.1256688 25430773

[B49] Tohtirjap A, Hou SX, Rivoire B, Gates G, Wu F, Dai YC (2023) Two new species of *Exidia**sensu lato* (*Auriculariales*, *Basidiomycota*) based on morphology and DNA sequences. Frontiers in Microbiology 13: 1080290. 10.3389/fmicb.2022.1080290 PMC9973447 36866163

[B50] Vilgalys R, Hester M (1990) Rapid genetic identification and mapping of enzymatically amplified ribosomal DNA from several *Cryptococcus* species. Journal of Bacteriology 172(8): 4238–4246. 10.1128/jb.172.8.4238-4246.1990 PMC213247 2376561

[B51] Wang K, Zhao MJ, Cai L (2025) Annual review on nomenclature novelties of fungi in China and around the world (2024). Biodiversity Science 33: 25355. 10.17520/biods.2025355

[B52] Wang S, Thorn RG (2021) *Exidia qinghaiensis*, a new species from China. Mycoscience 62(3): 212–216. 10.47371/mycosci.2021.03.002 PMC9157777 37091320

[B53] Wang XW, Liu SL, Zhou LW (2023) An updated taxonomic framework of *Hymenochaetales* (*Agaricomycetes*, *Basidiomycota*). Mycosphere 14: 452–496. 10.5943/mycosphere/14/1/6

[B54] Weiss M, Oberwinkler F (2001) Phylogenetic relationships in *Auriculariales* and related groups–hypotheses derived from nyclear ribosomal DNA sequences. Mycological Research 105: 403–415. 10.1017/S095375620100363X

[B55] Wells K, Bandoni RJ (2001) *Heterobasidiomycetes*, In: McLaughlin DJ, McLaughlin EG, Lemke PA (Eds) The Mycota. (A Comprehensive Treatise on *Fungi* as Experimental Systems for Basic and Applied Research), vol 7B. Springer, Berlin, Heidelberg, 85–120. 10.1007/978-3-662-10189-6_4

[B56] Wells K, Bandoni RJ, Lim SR, Berbee ML (2004) Observations on some species of *Myxarium* and reconsideration of the *Auriculariaceae* and *Hyaloriaceae (Auriculariales)*. IHW-Verlag, Germany.

[B57] White TJ, Bruns T, Lee S, Taylor J (1990) Amplification and direct sequencing of fungal ribosomal RNA genes for phylogenetics. In: Innis MA, Gelfand DH, Sninsky JJ, White TJ (Eds), PCR protocols: A guide to methods and applications. Academic Press, San Diego 315–322. 10.1016/B978-0-12-372180-8.50042-1

[B58] Wu F, Zhao Q, Yang ZL, Ye SY, Rivoire B, Dai YC (2020) *Exidia yadongensis*, a new edible species from East Asia. Mycosystema 39: 1203–1214. 10.13346/j.mycosystema.200205

[B59] Wu F, Tohtirjap A, Fan LF, Zhou LW, Alvarenga RLM, Gibertoni TB, Dai YC (2021) Global diversity and updated phylogeny of *Auricularia* (*Auriculariales*, *Basidiomycota*). Journal of Fungi 7(11): 933. 10.3390/jof7110933 PMC8625027 34829220

[B60] Xu Y, Yang Y, Yang X, Chen DX, Zheng W, Shen KZ, Zhang SC, Zhao CL (2025) Molecular phylogeny and taxonomy reveal two new genera and five new species in *Phanerochaetaceae (Polyporales)* from Yunnan, Southwest China. MycoKeys 113: 263–294. 10.3897/mycokeys.113.140624 PMC11840430 39980721

[B61] Yang Y, Xu Y, Wang L, Jiang QQ, Su JQ, Li R, Zhou HM, Zhao CL (2025) Multigene phylogeny of seven wood-inhabiting fungal orders in *Basidiomycota*, and proposal of a new genus and thirteen new species. Mycosphere 16(1): 245–295. 10.5943/mycosphere/16/1/4

[B62] Yuan HS, Lu X, Decock C (2018) Molecular and morphological evidence reveal a new genus and species in *Auriculariales* from tropical China. MycoKeys 35: 27–39. 10.3897/mycokeys.35.25271 PMC6021479 30622400

[B63] Yuan HS, Zhou LJ, Zhu YQ, Wei YL, Zhang XJ, Zhao CL, Zhao H (2026) Species diversity of corticioid and hydnoid fungi in China and their medicinal, environmental, agricultural and industrial values. Mycosphere 11: 287–1302.

[B64] Zhao CL, Qu MH, Huang RX, Karunarathna SC (2023) Multi‐gene phylogeny and taxonomy of the wood‐rotting fungal genus *Phlebia**sensu lato* (*Polyporales*, *Basidiomycota*). Journal of Fungi (Basel, Switzerland) 9(3): 320. 10.3390/jof9030320 PMC10058843 36983488

[B65] Zhou LW, Dai YC (2013) Phylogeny and taxonomy of poroid and lamellate genera in the *Auriculariales (Basidiomycota)*. Mycologia 105: 1219–1230. 10.3852/12-212 23709572

